# Sphingosine kinase 2 suppresses neutrophil responses to promote viral persistence while attenuating immune pathology

**DOI:** 10.3389/fimmu.2025.1706967

**Published:** 2026-01-22

**Authors:** Vijayamahantesh Vijayamahantesh, Ying He, Lei Jiang, Hailey Huerter, Kwang Il Jung, Savannah McKenna, Caleb J. Studstill, Lee-Ann H. Allen, Ravi Nistala, Dong Xu, Bumsuk Hahm

**Affiliations:** 1 Departments of Surgery and Molecular Microbiology and Immunology, University of Missouri, Columbia, MO, United States; 2 Department of Electrical Engineering and Computer Science, and Christopher S. Bond Life Sciences Center, University of Missouri, Columbia, MO, United States; 3 Department of Molecular Microbiology and Immunology, University of Missouri, Columbia, MO, United States; 4 Division of Nephrology, Department of Medicine, University of Missouri, Columbia, MO, United States

**Keywords:** immune suppression, neutrophil regulation, sphingosine kinase, T cell regulation, virus persistence

## Abstract

Chronic viral infections often suppress immune cell functions, which helps restrict immune pathology but leads to viral persistence. However, the underlying mechanisms are not completely understood. We recently found that sphingosine kinase 2 (SphK2)-deficient (*Sphk2*^−/−^) mice succumb to lymphocytic choriomeningitis virus (LCMV) infection due to immune pathology. In addition to heightened T cell immunity, a notable increase in neutrophil numbers was observed in LCMV-infected *Sphk2*^−/−^ mice. Depletion of neutrophils increased the viability of virus-infected *Sphk2*^−^*^/^*^−^ mice, supporting the role of SphK2-deficient neutrophils in viral immune pathogenesis. Furthermore, SphK2-deficient neutrophils expressed lower levels of the immunosuppressive marker CD244 during infection. Importantly, adoptively transferred SphK2-deficient neutrophils demonstrated intrinsic regulation of CD244 and improved virus-specific T cell responses, resulting in a diminished viral burden. Transcriptomic analysis revealed an increased expression of pro-inflammatory and antiviral genes in SphK2-deficient neutrophils. These results indicate that SphK2 promotes suppressive neutrophil responses and regulates neutrophil-associated immune pathology during persistent infections. Our findings may help in the design of new immunotherapeutics to control chronic viral diseases.

## Introduction

Viruses can establish persistent infections, causing chronic and fatal illnesses that account for almost one million deaths per year (1). Chronic viral infections often induce immunosuppressive conditions by regulating diverse immune cells and factors. Viral immune suppression is exemplified by the gradual loss of effector T cell function (2). However, the molecular and cellular mechanisms underlying immune dysfunction during chronic infections are not completely understood.

Lymphocytic choriomeningitis virus (LCMV) infection in mice has been useful for studying the host immune response to infections and understanding key immunological mechanisms (3–9). The LCMV prototypic strain Armstrong (Arm) causes acute infection, as the virus elicits a potent CD8^+^ effector T cell response that results in subsequent viral clearance within a week. In contrast, LCMV clone 13 (Cl 13), a variant of the parental Arm strain, induces immune suppression, allowing the virus to persist for up to 60–100 days post-infection (10–13). Mouse models using LCMV Cl 13 have also been instrumental in uncovering immunological concepts such as virus-specific T cell exhaustion (12–15). These concepts were true for other chronic infections and diseases in humans and significantly contributed to the development of the first immunotherapy targeting PD-1 for the treatment of cancer (16).

Sphingolipids are bioactive lipid molecules that are not only essential structural components of the lipid bilayer but also key players in intra- and extracellular signaling systems (17). Sphingosine 1-phosphate (S1P) is a sphingolipid metabolite and an important regulator of inflammation and immune response (18, 19). Generation of S1P from sphingosine is catalyzed by sphingosine kinase 1 (SphK1) and sphingosine kinase 2 (SphK2). These two enzymes are encoded by two genes and differ in their subcellular localization, substrate specificity, and function. SphK1 is primarily localized in the cytosol and translocates to the plasma membrane upon activation (20). S1P produced by SphK1 on the plasma membrane acts in an autocrine or paracrine manner to promote cell survival and proliferation. Although SphK2 is found in the cytoplasm, it shuttles between the nucleus and other subcellular organelles and regulates epigenetic gene expression (21). Despite sharing enzymatic activities, SphK1 and SphK2 differentially regulate immune responses through mechanisms that are not well defined (22).

We previously showed that deletion of SphK2 increased T cell response, immune pathogenesis, and eventually death of LCMV Cl 13-infected mice due to vascular leakage in the kidneys (23). Notably, we also observed a significantly increased neutrophil population in the kidneys of LCMV Cl 13-infected SphK2-deficient mice compared to their wild-type (WT) counterparts. Historically, neutrophils have been considered sentinels of the immune system and the first cells to reach the site of infection or inflammation. However, recent studies have demonstrated that neutrophils can function in diverse ways, exhibit transcriptional and functional plasticity in response to extracellular cues, and participate in shaping innate and adaptive immune responses (24). Myeloid cells, such as myeloid-derived suppressor cells (MDSCs), play a key role in T cell exhaustion, leading to the development of virus persistence (25–27). However, the mechanism of immunosuppression by myeloid cells, especially neutrophils, remains unclear.

In this study, we investigated the role of neutrophils in chronic LCMV infection. Our results demonstrate that SphK2 intrinsically regulates the expression of the immune regulatory molecule CD244 on neutrophils during infection, and SphK2-deficient neutrophils exhibit immune stimulatory effects on T cells. Single-cell RNA sequencing (scRNA-seq) of bone marrow neutrophils (BMNs) revealed the role of SphK2 in regulating several key genes required for the development of immune suppressive neutrophils. Taken together, these results suggest that SphK2 is a valuable therapeutic target for chronic infections and inflammatory conditions.

## Results

### SphK2 deficiency induces expansion of neutrophils upon LCMV Cl 13 infection which contributes to viral immune pathogenesis

**Figure 1:**
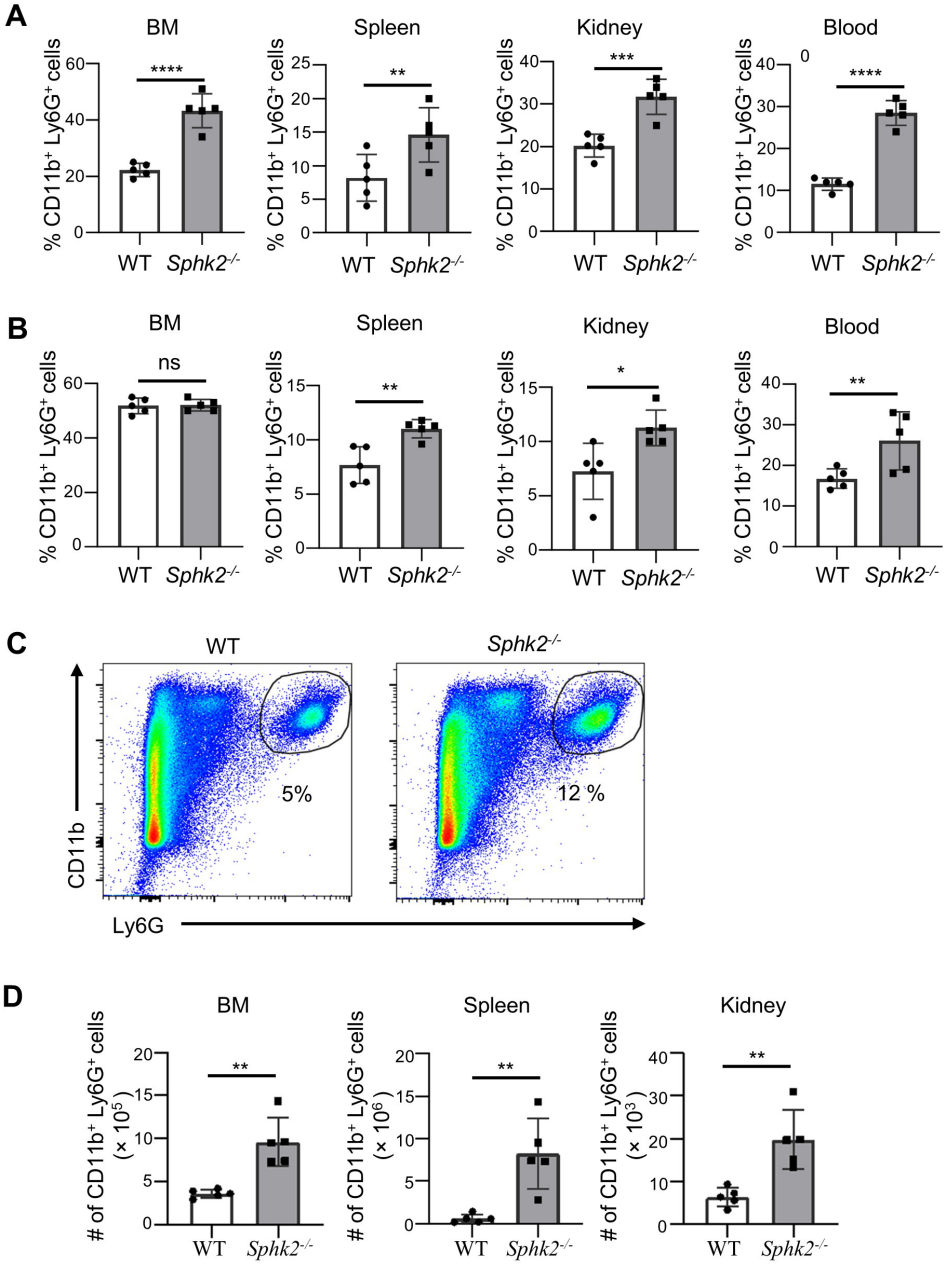
Deletion of *Sphk2* induces an increase in neutrophils upon LCMV Cl 13 infection. WT and *Sphk2^−/−^* mice (n = 5/group) were infected with LCMV Cl 13. Mice were sacrificed at 3 dpi and 8 dpi, and organs were collected. The percentage of neutrophils (CD11b^+^Ly6G^+^) in bone marrow (BM), spleen, blood, and kidney of LCMV Cl 13 infected WT and *Sphk2^−/−^* mice at 3 dpi **(A)** and 8 dpi **(B)** was quantified using flow cytometry. A representative flow cytometry data of splenic neutrophils from C57BL/6 WT and *Sphk2^−/−^* mice at 3 dpi is shown **(C)**. Absolute number of neutrophils in BM, spleen, and kidney was assessed at 3 dpi **(D)**. ****p ≤0.0001, ***p ≤0.001, **p ≤0.01, *p ≤0.05, ns, not significant, bidirectional, unpaired Student’s *t*-test. Data are representative of two to three independent experiments.

LCMV Cl 13 infection of SphK2-deficient mice resulted in increased T cell responses associated with immune pathology in the kidneys and eventually led to the death of infected mice. While SphK2 deficiency scarcely affected other immune cell components, we observed increased neutrophil infiltration into the kidneys of *Sphk2^−/−^* mice compared to that in wild-type (WT) mice upon infection (23). As neutrophils are often responsible for inflammation and tissue damage, neutrophil responses in *Sphk2^−/−^* mice were further assessed during Cl 13 infection. The percentage and distribution of neutrophils can change rapidly during infection, affecting the establishment of inflammation and immune responses (28). We assessed the changes in neutrophil populations during chronic LCMV infection. As shown in **Figure 1**, the neutrophil counts in the spleen, blood, and kidneys of *Sphk2^−/−^* mice were significantly higher than those in WT mice at 3 days post-infection (dpi) (**Figure 1A**) and 8 dpi (**Figure 1B**). The neutrophil surge in the spleen, blood, and kidneys of *Sphk2^−/−^* mice at 3 dpi was in line with the significantly increased neutrophil frequency in the BM of *Sphk2^−/−^* mice (**Figures 1A, C**). The number of SphK2-deficient neutrophils also increased in the BM, spleen, and kidneys (**Figure 1D**). Furthermore, unlike neutrophils, CD11b^+^Ly6C^+^ monocytes and F4/80^+^CD11b^+^ macrophage populations did not increase in the blood of *Sphk2^−/−^* mice at 3 dpi (**Supplementary Figure S1**). Interestingly, at 8 dpi, although there was no significant difference in the frequency of neutrophils in the BM, we observed a significant increase in the frequency of neutrophils in the blood, spleen, and kidney (**Figure 1B**). The increase in neutrophil frequencies in the spleen and blood was sustained when assessed at 14 dpi (**Supplementary Figure S2**). These results indicate that LCMV Cl 13 induces early expansion of neutrophils in *Sphk2^−/−^* mice, and that this increased number of neutrophils persists during the infection.

**Figure 2:**
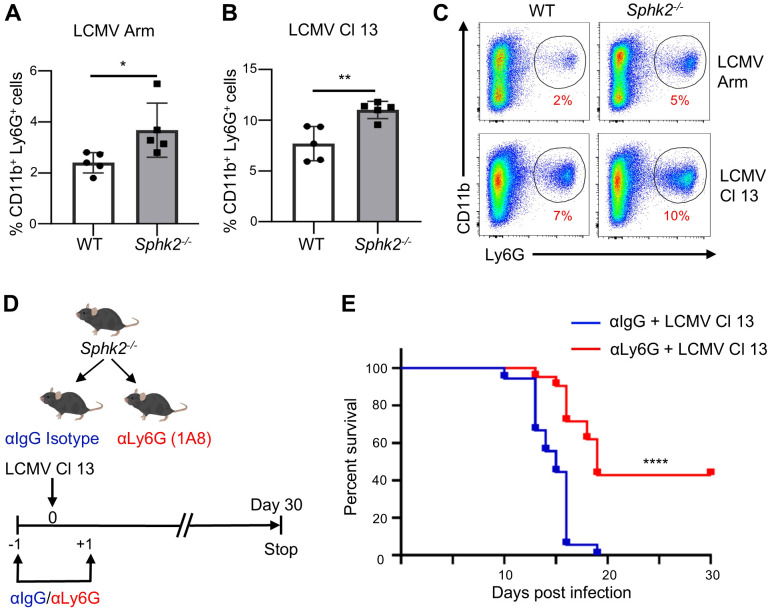
Increased neutrophil expansion by SphK2 deficiency is independent of LCMV strains and contributes to Cl 13-induced mortality of *Sphk2^−^*^/^*^−^* mice. WT and *Sphk2^−/−^* mice (n = 5/group) were infected with LCMV Arm or Cl 13. At 10 dpi, the percentage of neutrophils (CD11b^+^Ly6G^+^) in the spleen of mice infected with LCMV Arm **(A)** and Cl 13 **(B)** was quantified by flow cytometry. A representative flow cytometry data of splenic neutrophils from WT and *Sphk2^−/−^* mice at 10 dpi is shown **(C)**. Depletion of neutrophils was performed by injecting *Sphk2^−/−^* mice with the 250 µg anti-Ly6G antibody (αLy6G) or control-antibody (αIgG) via the intraperitoneal (i.p.) route one day before and one day after LCMV Cl 13 infection. The flow diagram of the neutrophil depletion strategy is shown in **(D)**. The comparison of survival of isotype antibody (n = 18) and anti-Ly6G (n = 21) treated *Sphk2^−/−^* mice upon LCMV Cl 13 infection was monitored for 30 days **(E)**. ****p ≤0.0001, **p ≤0.01, *p ≤0.05, bidirectional, unpaired Student’s *t*-test for **(A)** and **(B)** and Kaplan–Meier test for **(E)**.

To determine whether the increased neutrophil expansion observed in LCMV Cl 13-infected *Sphk2^−/−^* mice was an LCMV Cl 13 strain-specific event, *Sphk2^−/−^* and WT mice were infected with LCMV Arm or Cl 13. On day 10 post-infection, the neutrophil population in the spleens of infected mice was analyzed. We found a significantly increased frequency of neutrophils in *Sphk2^−/−^* mice compared to WT mice, irrespective of the strains used (**Figures 2A–C**). These results suggest that neutrophil expansion is regulated by SphK2, regardless of the LCMV strain used. However, LCMV Cl 13 infection induced the accumulation of a higher percentage of neutrophils than LCMV Arm infection (**Supplementary Figures S3A, B**), implying that neutrophils may play a role in immune regulation during Cl 13 infection.

The systemic increase in neutrophils observed in LCMV Cl 13-infected *Sphk2^−/−^* mice led us to hypothesize that neutrophils contribute to the immune pathology of kidney damage and the ultimate mortality of LCMV Cl 13-infected *Sphk2^−/−^* mice. To test this hypothesis, we adopted a neutrophil depletion strategy during LCMV Cl 13 infection. Using anti-Ly6G (1A8) antibody (αLy6G), neutrophils were depleted one day before and one day after LCMV Cl 13 infection, followed by monitoring of mouse mortality, as shown in **Figure 2D**. Antibody-mediated neutrophil depletion was confirmed at 3 dpi and 7 dpi (**Supplementary Figure S4**). Depletion of neutrophils significantly increased the survival of Cl 13-infected *Sphk2^−/−^* mice compared to that of the isotype control antibody (αIgG)-treated group (**Figure 2E**). The failure to completely rescue LCMV Cl 13-infected *Sphk2^−/−^* mice by neutrophil depletion suggests the potential involvement of other immune cells, such as T cells, which are critical for virus-induced immune pathology (23). Taken together, these results indicate that SphK2 deficiency induces neutrophil expansion, which contributes to immune pathogenesis.

### SphK2 intrinsically regulates CD244 expression on neutrophils upon LCMV Cl 13 infection

**Figure 3:**
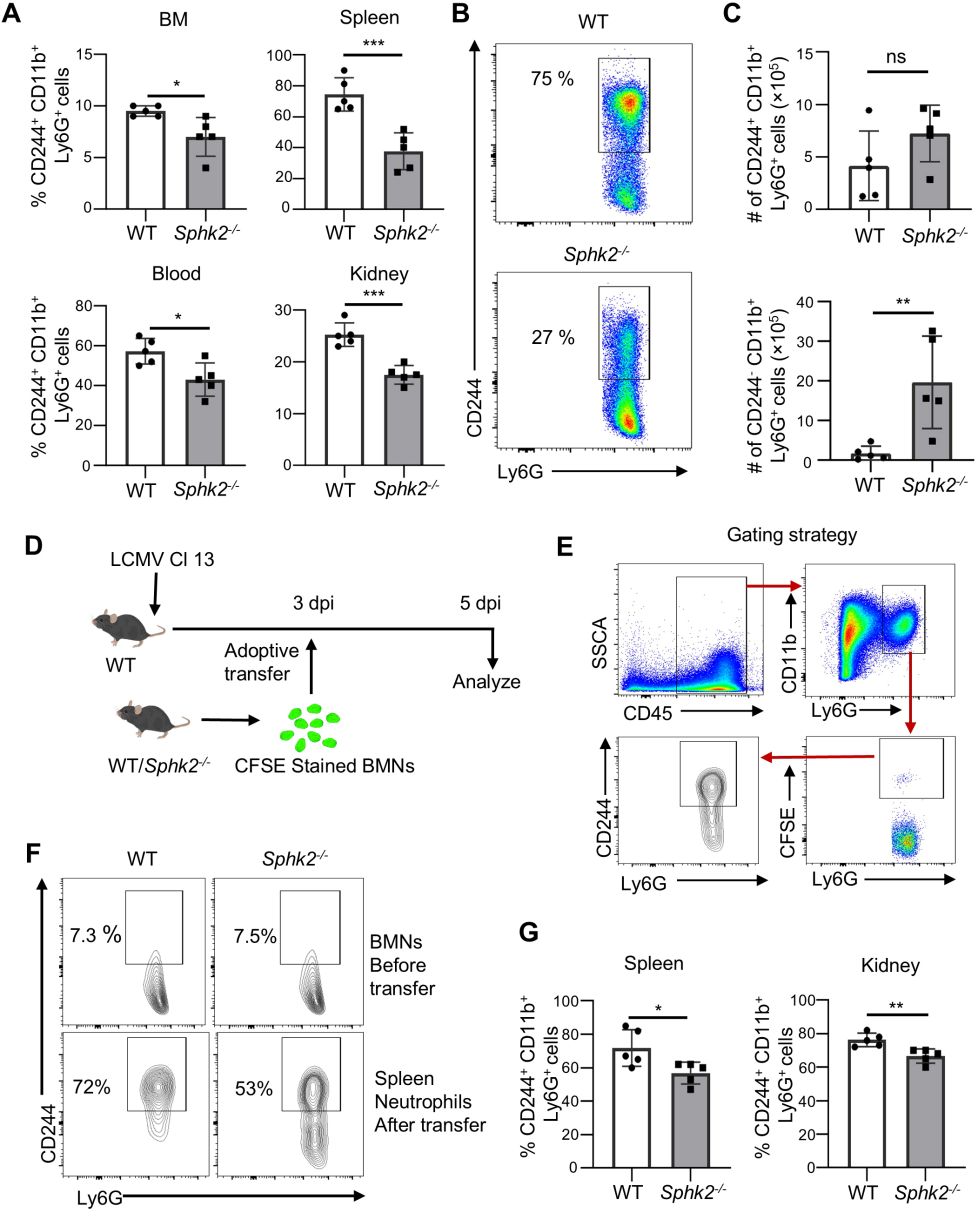
Expression of CD244 is significantly reduced on *Sphk2^−/−^* neutrophils and is intrinsically regulated by SphK2. WT and *Sphk2^−^*^/^*^−^* mice (n = 5/group) were infected with LCMV Cl 13, and at 3 dpi, CD244^+^ neutrophils in organs were measured by flow cytometry **(A)**. A representative flow cytometry data of CD244^+^ neutrophils between WT and *Sphk2^−/−^* mice on 3 dpi is shown **(B)**. Absolute numbers of CD244^+^ and CD244*^−^* neutrophils in spleen were assessed at 3 dpi **(C)**. To determine the intrinsic regulation of CD244 expression by SphK2 on neutrophils, the experiments were performed by using adoptive transfer of donor BMNs **(D–G)**. Naïve BMNs were isolated from WT and *Sphk2^−^*^/^*^−^* mice (n = 5/group), stained with CFSE, and then adoptively transferred to LCMV Cl 13-infected WT mice at 3 dpi. The transferred cells were detected in the spleen and kidney at 5 dpi by flow cytometry. The flow diagram of neutrophil adoptive transfer **(D)** and the gating strategy of detecting CFSE-stained donor neutrophils in recipient mice **(E)** are depicted. Representative CD244^+^ neutrophil in donor BMNs before transfer (top) and on donor cells in the recipient spleen after transfer (bottom) are shown **(F)**. The percentage of CD244 expression on CFSE-stained donor cells in the spleen and kidney of recipient mice was assessed **(G)**. ***p ≤0.001, **p ≤0.01, *p ≤0.05, ns. not significant. bidirectional, unpaired Student’s *t*-test.

Although the lack of specific surface markers makes it challenging to separate neutrophil subpopulations, a few studies have exploited CD244 to differentiate suppressive neutrophils from resting or immune-stimulatory neutrophils (29–31). As a member of the signaling lymphocyte activation molecule family (SLAMF) receptors, CD244 can transmit an inhibitory or activation signal in lymphocytes but generally transmits an inhibitory signal in myeloid cells due to the differential downstream regulatory pathways (32). Following LCMV Cl 13 infection, CD244 was upregulated on the surface of neutrophils in the BM, spleen, blood, and kidneys of WT mice compared to the uninfected group (**Supplementary Figure S5**). To determine whether SphK2 affects the expression level of CD244 on neutrophils during Cl 13 infection, CD244 expression on neutrophils (CD244^+^CD11b^+^Ly6G^+^ cells) was assessed in multiple organs of WT and *Sphk2^−/−^* mice at 3 dpi. The expression of CD244 on neutrophils and the percentage of CD244^+^ neutrophils significantly decreased in the BM, spleen, blood, and kidney of *Sphk2^−/−^* mice compared to WT mice (**Supplementary Figure S6**, **Figure 3A**). The representative plots show a clear difference in CD244^+^Ly6G^+^ cells between WT and *Sphk2^−^*^/^*^−^*mice (75% vs. 27%) (**Figure 3B**). Although the number of CD244^+^Ly6G^+^ cells did not significantly change, presumably due to the increase in the total number of SphK2-deficient neutrophils, the number of CD244-deficient neutrophils (CD244^-^Ly6G^+^) substantially increased in *Sphk2^−/−^* mice upon infection (**Figure 3C**). These results indicate that SphK2 increases CD244 expression on neutrophils upon LCMV Cl 13 infection.

To investigate whether SphK2 can regulate CD244 expression in a neutrophil-intrinsic manner under LCMV infection conditions, CFSE-stained BM neutrophils (BMN) from uninfected WT or *Sphk2^−^*^/^*^−^* mice were adoptively transferred into LCMV Cl 13-infected WT mice on day 3 post-infection (**Figure 3D**). The expression of CD244 on donor neutrophils (CFSE stained) in the spleen and kidneys of recipient mice was assessed (**Figures 3E, F**). Prior to adoptive transfer, the basal level of CD244^+^ neutrophils in donor BMNs was approximately 7% and comparable between WT and *Sphk2^−^*^/^*^−^* mice (**Figure 3F**). However, when these neutrophils were exposed to the LCMV Cl 13 infection environment, the transferred WT neutrophils acquired high CD244 expression levels. Consistent with the *Sphk2^−/−^* phenotype, SphK2-deficient neutrophils in the infection microenvironment showed reduced CD244^+^ neutrophils compared to those from WT donor mice (72% vs. 53%, as shown in **Figures 3F, G**). These observations indicate that SphK2 can intrinsically regulate CD244 expression on neutrophils during LCMV infection.

### *Sphk2^−/−^* neutrophils produce significantly higher ROS upon LCMV Cl 13 infection

**Figure 4:**
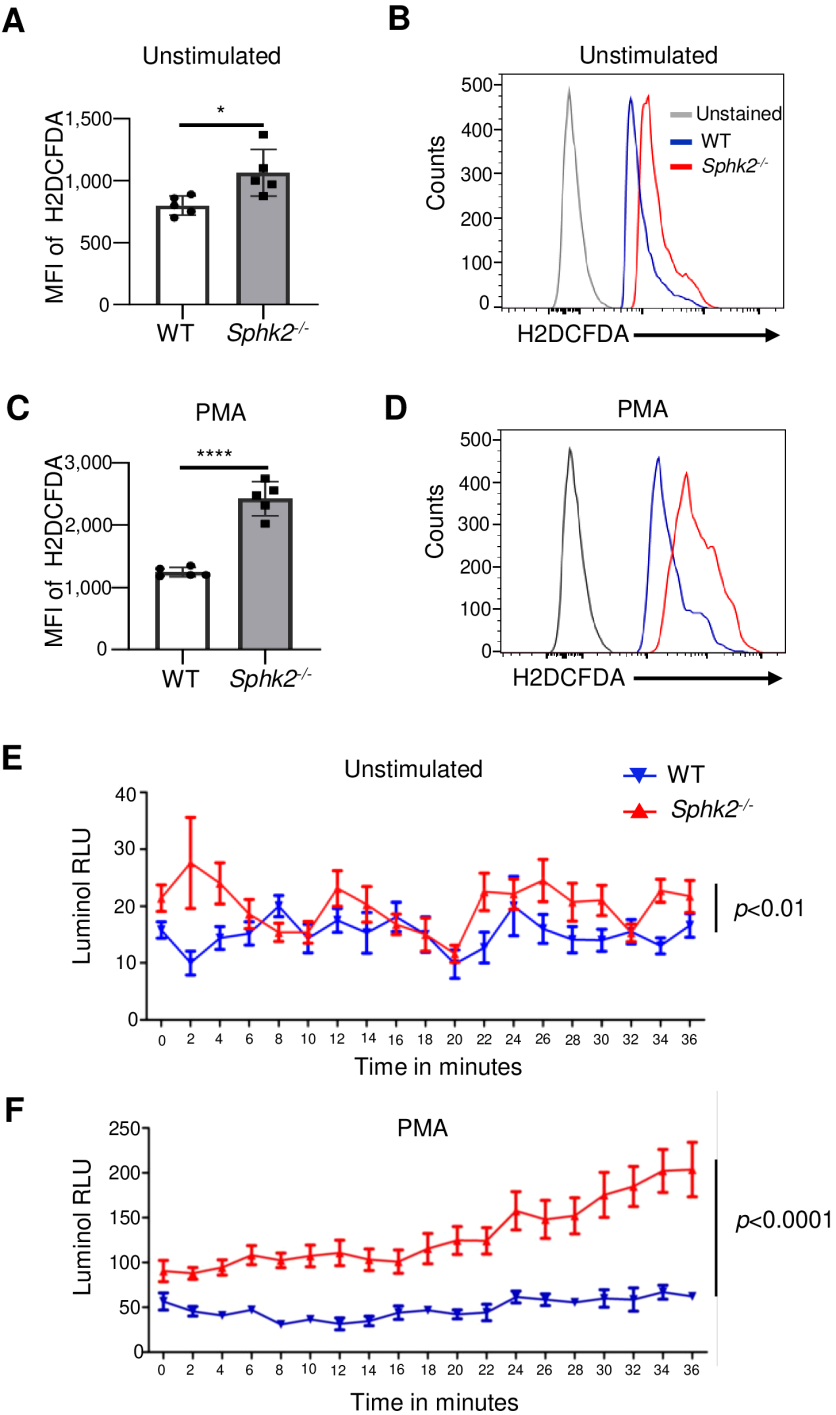
*Sphk2^−/−^* neutrophils produce significantly higher ROS upon LCMV Cl 13 infection. BMNs were isolated from LCMV Cl 13 infected WT and *Sphk2^−^*^/^*^−^* mice (n = 5/group) at 3 dpi and cultured in the absence **(A, B)** or presence of 50 nM PMA for 30 min at 37°C CO_2_ incubator **(C, D)**. To detect the intracellular ROS, neutrophils were incubated with H2DCFDA intracellular ROS detection probe **(A–D)** at 4°C for 30 min in dark. The mean fluorescence intensities (MFIs) of H2DCFDA were detected by flow cytometry **(A, C)** and representative histograms are shown **(B, D)**. Production of intracellular ROS was monitored in a time-dependent manner by the luminol method in the absence **(E)** or presence of 50 nM PMA **(F)**. ****p ≤0.0001, *p ≤0.05, unpaired t test performed for **(A)** and **(C)** and 2-way ANOVA for **(E)** and **(F)**.

Neutrophil production of reactive oxygen species (ROS) is a mechanism of tissue damage that contributes to disease severity (33). Therefore, we determined whether SphK2 deficiency alters the capacity of neutrophils to produce ROS by comparing the levels of ROS in BMNs of *Sphk2^−/−^* and WT mice following LCMV infection. ROS production was quantified by flow cytometry using the intracellular ROS detection probe H2DCFDA. Without LCMV infection, there was no significant difference in the basal level of ROS between WT and SphK2-deficient neutrophils in the presence or absence of PMA stimulation (**Supplementary Figures S7A, B**). However, upon LCMV infection, we found a significantly increased production of ROS by unstimulated neutrophils (**Figures 4A, B**) as well as PMA-stimulated neutrophils (**Figures 4C, D**) from *Sphk2^−/−^* mice compared to WT mice. ROS production was quantified in a time-dependent manner using luminol-enhanced chemiluminescence. In agreement with the results of flow cytometry, ROS production significantly increased in a time-dependent manner upon LCMV infection of SphK2-deficient BMNs, both without (**Figure 4E**) and without PMA stimulation (**Figure 4F**). These data suggest that SphK2-deficient neutrophils are significantly activated and likely less immune suppressive during LCMV infection, which is in line with the reduced CD244 surface expression (**Figure 3**).

### Depletion of neutrophils during chronic LCMV infection improves virus-specific T cell functions and enhances virus clearance

**Figure 5:**
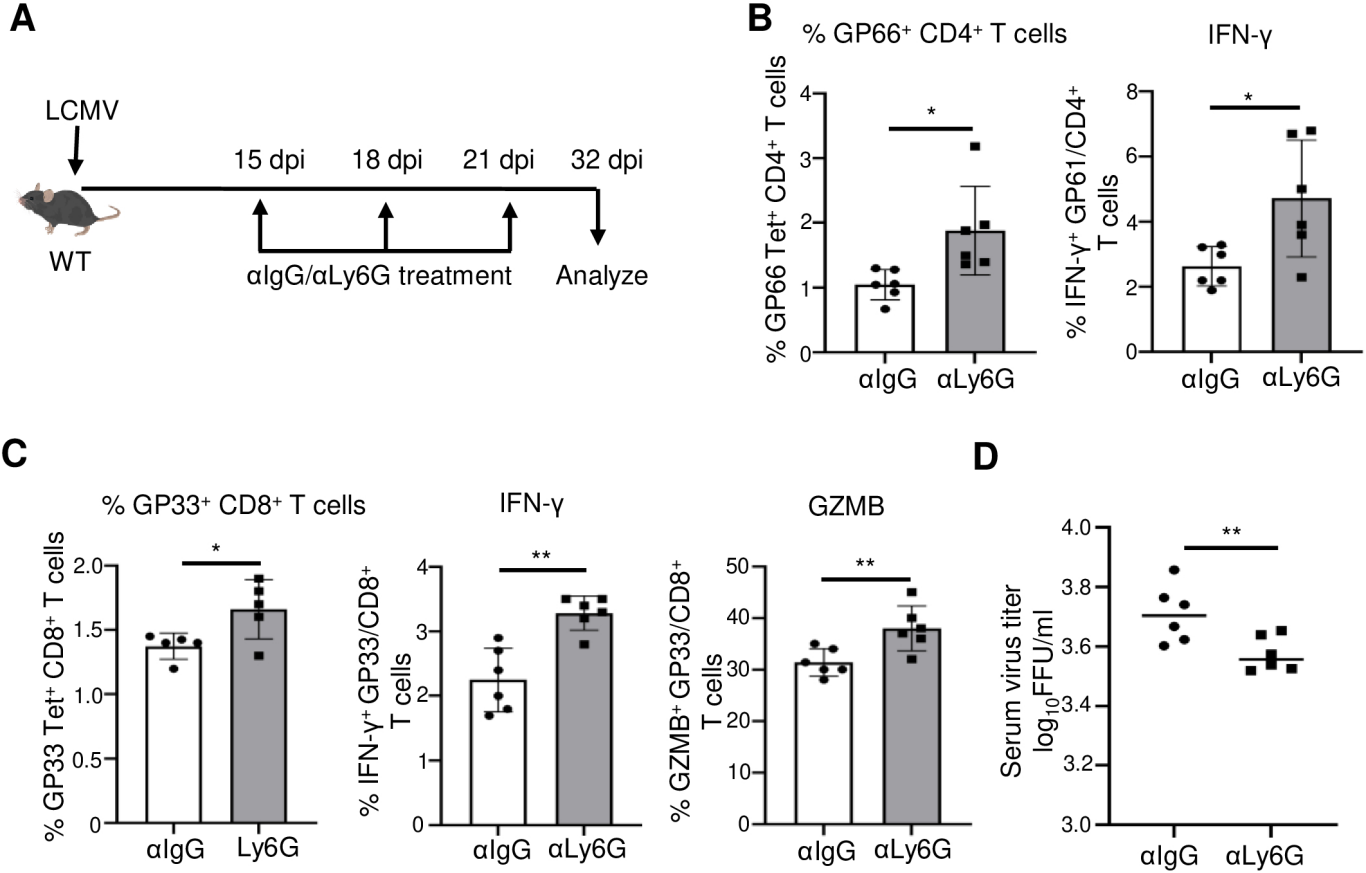
Depletion of neutrophils during chronic virus infection restores virus-specific T cell functions and enhances virus clearance. WT mice (n = 5–6/group) were infected with LCMV Cl 13, and neutrophils were depleted by injecting mice with 250 µg anti-Ly6G (clone 1A8) antibody via the i.p. route at 15 dpi, 18 dpi, and 21 dpi **(A)**. On day 32, splenocytes were harvested. The percentage of virus-specific GP_66–77_ (GP66) tetramer^+^CD4^+^ T cells and production of intracellular IFN-γ from splenic CD4^+^ T cells were assessed upon stimulation by peptides GP_61–80_ (GP61) **(B)**. The percentage of virus-specific GP_33–41_ (GP33) tetramer^+^CD8^+^ T cells in the spleen and production of intracellular IFN-γ and granzyme B (GZMB) upon stimulation by GP33 peptide **(C)** from CD8^+^ T cells were analyzed. The virus titer in the serum at 32 dpi was measured by FFU assay **(D)**. **p ≤0.01, *p ≤0.05, bidirectional, unpaired Student’s *t*-test.

Neutrophils can interact with various immune cells, including T cells, and orchestrate their activation, proliferation, and differentiation under certain conditions (24, 34). To reveal the role of neutrophils during chronic LCMV infection, we depleted neutrophils using αLy6G treatment on days 15, 18, and 21 post-infection, followed by analysis of antiviral T cell responses and virus clearance (**Figure 5A**). Flow cytometry analysis showed that αLy6G treatment efficiently depleted neutrophils (**Supplementary Figure S8**). Functionally, neutrophil depletion enhances the antiviral T cell response. Depletion resulted in increased IFN-γ production and increased numbers of LCMV GP_66–77_ (GP66)-specific CD4^+^ T cells (GP66 tetramer^+^CD4^+^ T cells) (**Figure 5B**). Similarly, we observed significantly increased LCMV GP_33–41_ (GP33)-specific CD8^+^ T cells (GP33 tetramer^+^CD8^+^ T cells) and intracellular IFN-γ and granzyme B (GZMB) production from CD8^+^ T cells (**Figure 5C**). The increase in T cell functions following treatment with anti-Ly6G antibody was associated with significantly accelerated viral clearance in the serum compared to that in the isotype control treatment (**Figure 5D**). Collectively, these results confirm that neutrophils exert an immunosuppressive effect on virus-specific T cells, which helps promote LCMV persistence.

### Adoptively transferred *Sphk2*^−/−^ neutrophils promote LCMV-specific T cell responses

**Figure 6:**
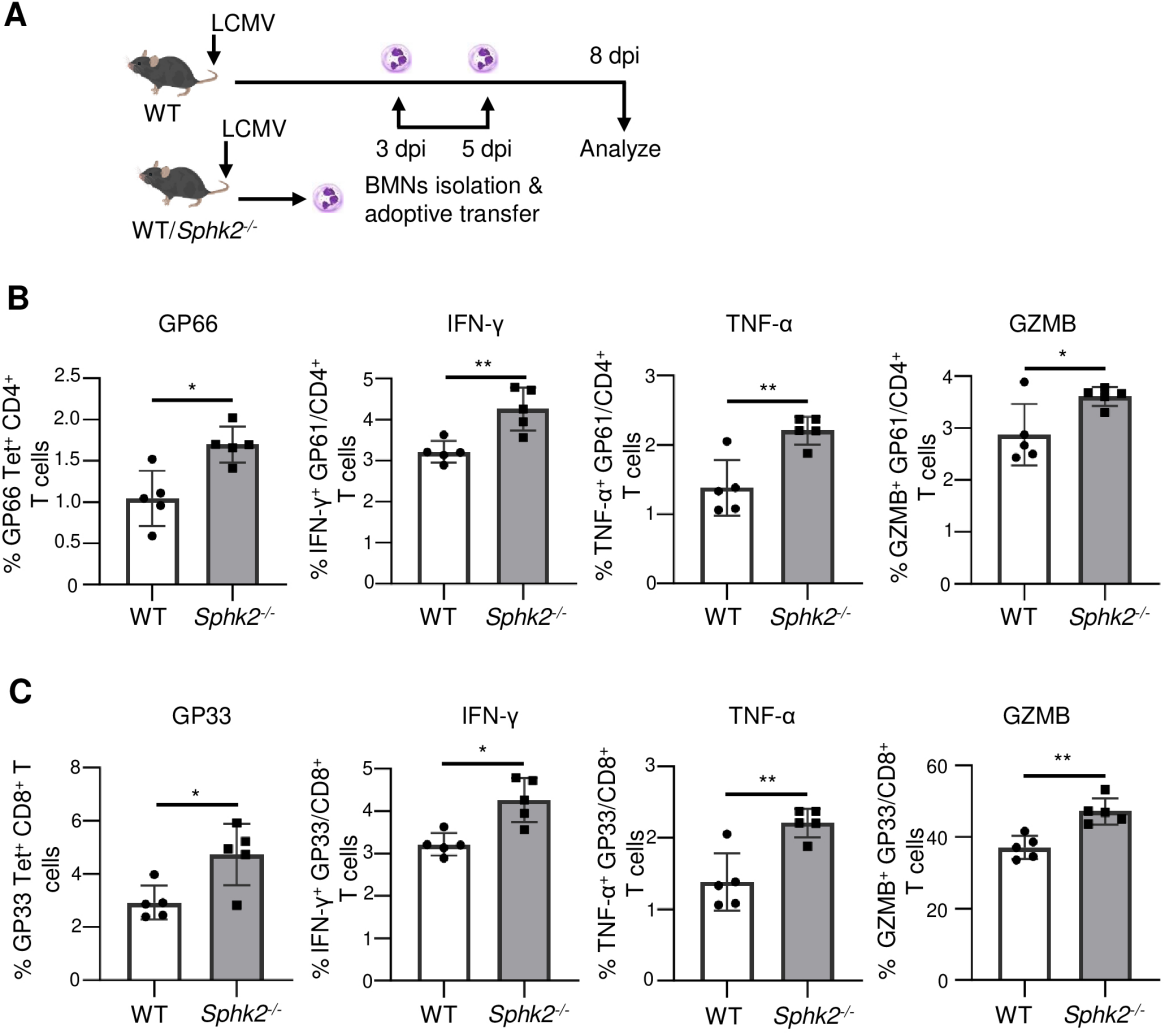
Adoptive transfer of *Sphk2^−/−^* neutrophils significantly improves the T cell responses to LCMV infection. BMNs were isolated from LCMV Cl 13-infected WT or *Sphk2^-/-^* mice (n = 5) at 3 dpi and then adoptively transferred to LCMV Cl 13-infected recipient WT mice (n = 5/group) at 3 dpi and 5 dpi. The schematic presentation of the adoptive transfer strategy is depicted **(A)**. On day 8 post LCMV infection, splenocytes were collected from recipient mice and CD4^+^ and CD8^+^ T cells were *in vitro* re-stimulated with GP61 or GP33 peptide respectively. GP66 Tet^+^CD4^+^ T cells **(B)** and GP33 Tet^+^CD8^+^ T cells **(C)** were assessed for their production of IFN-γ, TNF-α, and GZMB. **p ≤0.01, *p ≤0.05, bidirectional, unpaired Student’s *t*-test.

These results demonstrate the immunosuppressive effect of neutrophils on virus-specific CD4^+^ and CD8^+^ T cells during chronic LCMV infection. However, SphK2-deficient neutrophils showed significantly decreased inhibitory receptors on their surface and produced significantly increased ROS when exposed to LCMV, indicating the pro-inflammatory functions of *Sphk2*^−^*^/^*^−^ neutrophils. To test whether SphK2-deficient neutrophils exert an immune regulatory effect on T cells during LCMV infection, we employed an adoptive transfer strategy (**Figure 6A**). BMNs were isolated from LCMV Cl 13-infected WT and Sphk2^−/−^ mice at 3 dpi. The comparable purities of the enriched WT and *Sphk2*^−^*^/^*^−^ BMNs were confirmed before transfer (**Supplementary Figure S9**). These BMNs were transferred to LCMV Cl 13-infected WT mice at 3 dpi and 5 dpi. On day 8 post-infection, LCMV antigen-reactive CD4^+^ and CD8^+^ T cell responses were analyzed in recipient mice. Mice that received SphK2-deficient neutrophils showed significantly increased frequencies of virus-specific CD4^+^ (**Figure 6B**) and CD8^+^ T cells (**Figure 6C**). Furthermore, the transfer of SphK2-deficient neutrophils significantly enhanced the functional properties of CD4^+^ and CD8^+^ T cells, that is, increased IFN-γ, TNF-α, and granzyme B production, compared to the T cells of mice that received WT neutrophils. T cell analyses were performed using antigenic re-stimulation with the peptides GP_61–80_ (GP61) (**Figure 6B**) or GP_33–41_ (GP33) (**Figure 6C**). These results demonstrate that *Sphk2*^−/−^ neutrophils exert an immune stimulatory effect on virus-specific T cells during LCMV infection *in vivo*.

**Figure 7:**
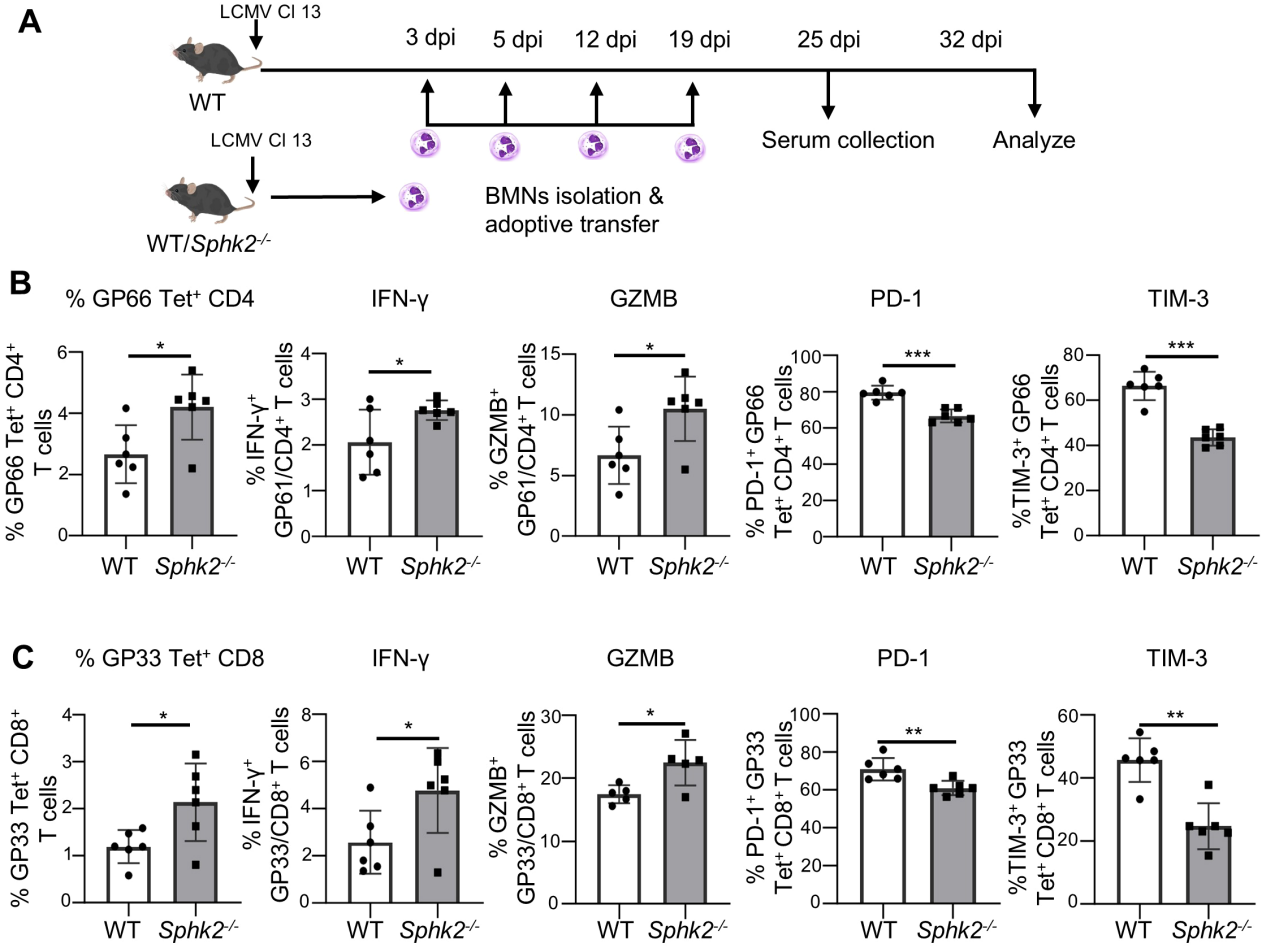
Adoptively transferred *Sphk2^−/−^* neutrophils improve LCMV-specific T cell responses. BMNs from LCMV Cl 13 infected WT or *Sphk2^−/−^* mice (n = 6) were adoptively transferred to LCMV Cl 13 infected recipient mice (n = 6/group) at multiple time points as shown in the workflow diagram **(A)**. The number of virus-specific T cells, intracellular IFN-γ and GZMB production, expression of PD-1 and TIM-3 on GP66 Tet^+^CD4^+^**(B)** and GP33 Tet^+^ CD8^+^ T cells **(C)** were measured by flow cytometry. ***p ≤0.001, **p ≤0.01, *p ≤0.05, bidirectional, unpaired Student’s *t*-test.

Next, we designed a similar longitudinal study to test whether these immunostimulatory effects are sustained and promote accelerated clearance of the virus. As depicted in **Figure 7A**, WT or *SphK2*^−/−^ neutrophils from LCMV-infected mice were adoptively transferred at 3 dpi, 5 dpi, 12 dpi, and 19 dpi, and T cell responses and virus titer were examined at 32 dpi. Similar to the previous experiment (8 dpi), we observed significantly increased GP66-specific CD4^+^ and GP33-specific CD8^+^ T cells, as well as significantly increased IFN-γ and granzyme B production at 32 dpi in SphK2-deficient neutrophil recipient mice (**Figures 7B, C**). This is supported by the significantly reduced exhaustion markers PD-1 and TIM-3 expression on the surface of both virus-specific CD4^+^ and CD8^+^ T cells (**Figures 7B, C**). Furthermore, the virus titer in the serum was modestly but significantly reduced at both 25 dpi and 32 dpi (**Supplementary Figures S10A, B**), and the virus titer in the liver and kidney also tended to decrease at 32 dpi in *Sphk2*^−/−^ neutrophil-recipient mice compared to WT neutrophil-recipient mice (**Supplementary Figures S10C, D**). These results indicate that SphK2-deficient neutrophils have immune stimulatory effects on T lymphocytes during chronic LCMV infection, which may help overcome T cell suppression and promote the elimination of LCMV from the serum and other organs.

### Innate pro-inflammatory and neutrophil activation-related genes are upregulated in *Sphk2^−/−^* neutrophils upon LCMV infection

**Figure 8:**
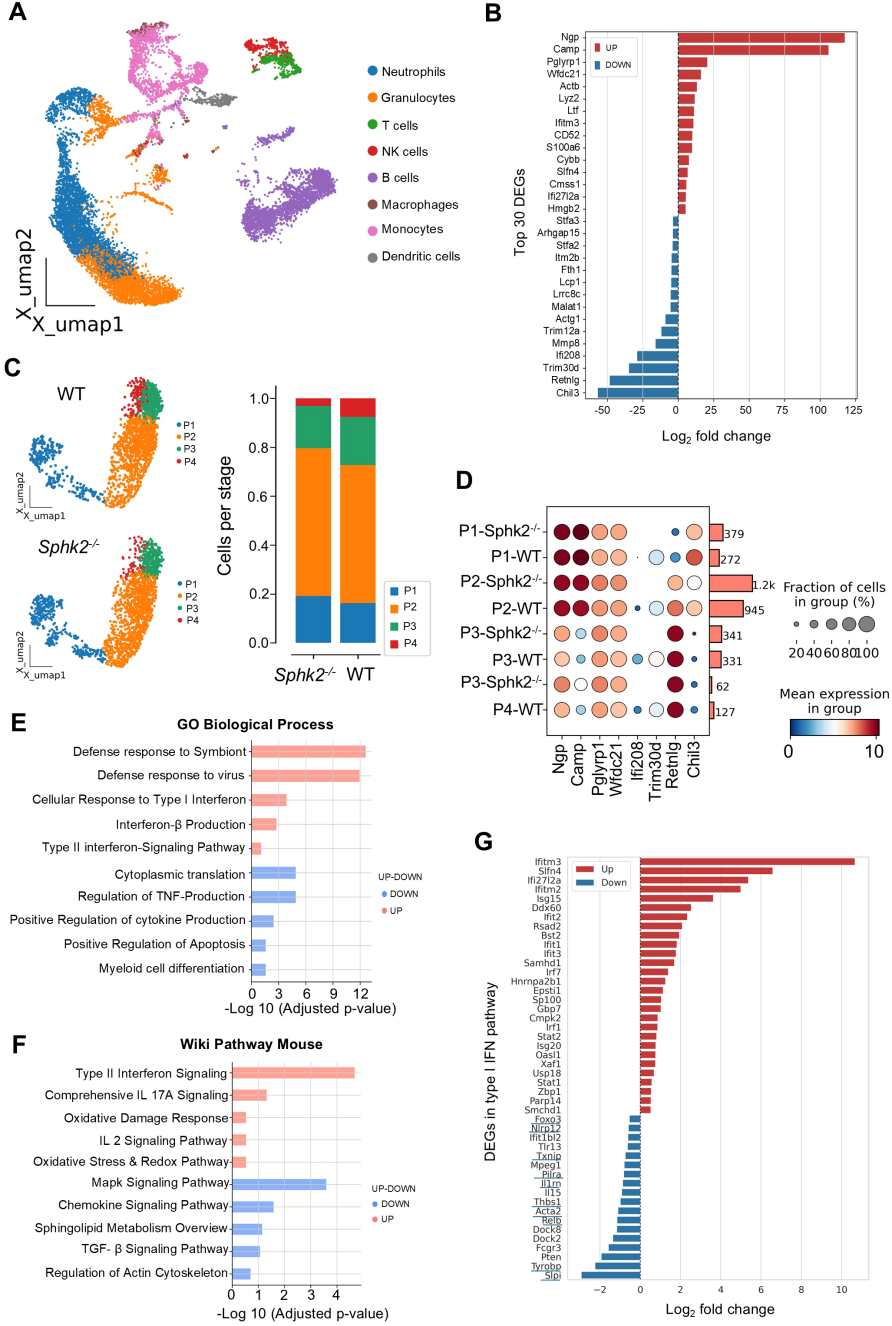
*Sphk2^−/−^* neutrophils from LCMV Cl 13 infected mice display a proinflammatory phenotype. WT and *Sphk2^−^*^/^*^−^* mice (n = 3/group) were infected by LCMV Cl 13, uninfected C57BL/6 WT mice (n = 3) were used as control. At 3 dpi bone marrows (BM) were collected, and BM cells were isolated and resuspended in PBS + 0.04% BSA at a density 1 × 10^6^ cells/ml. The cell suspensions were immediately sent to the DNA core facility for single-cell RNA-sequencing. An overview of UMAP of the cell cluster derived from BM of uninfected WT or infected WT and infected Sphk2*^−^*^/^*^−^* mice (n = 3), different color indicates distinct cell types **(A)**. The log_2_ fold changes for the top 30 differentially expressed genes (DEGs) that are upregulated or downregulated in *Sphk2^−/−^* mice **(B)**. UMAP of bone marrow neutrophil subclusters P1–P4 distribution and a bar graph showing the proportion of each neutrophil subcluster from *Sphk2^−/−^* and C57BL/6 WT mice **(C)**. Distribution of selected topmost DEGs on neutrophil subsets of WT and *Sphk2^−/−^* mice is shown, and cell events corresponding to different subsets are depicted by the bars on the right **(D)**. GO analysis of biological processes (BP) and Wiki pathways of up- and downregulated pathways in infected *Sphk2^−/−^* neutrophils compared to infected WT neutrophils **(E, F)**. The log_2_ fold changes for DEGs in type I IFN pathway that are upregulated or downregulated in neutrophils from *Sphk2^−/−^* mice are shown **(G)**. IFN-related genes that can limit excessive inflammation are underlined.

In response to infection, a large number of neutrophils are released into the circulation. However, different environmental cues can drive the development of distinct neutrophil subsets (24, 35). Changes in the neutrophil gene expression profile during the initial phase of infection are of great interest, as the accumulation of neutrophils at the site of infection and inflammation can alter the course of disease outcomes (36). Since SphK2-deficient BMNs isolated at 3 dpi displayed immune stimulatory activity, we next performed scRNA-seq of BM cells from WT and *Sphk2^−/−^* mice following 3 days of LCMV Cl 13 infection. The bone marrow cell types were annotated with a cell-specific standard gene expression profile (**Supplementary Figure S11**, **Figure 8A**). Based on these annotations, bone marrow neutrophils were selected for further gene expression and pathway analyses. A total of 972 transcripts were obtained from WT BMNs, of which 96 were found exclusively in the WT neutrophils. Similarly, we obtained 978 transcripts from *Sphk2^−/−^* BMNs, 126 of which were restricted to *Sphk2^−/−^* and not found in WT neutrophils. Based on the expression thresholds (1.5-fold increase or decrease in expression), we obtained 116 differentially expressed genes (DEGs) between WT and *Sphk2^−/−^* neutrophils. The top 30 DEGs included genes related to antimicrobial activity (*Ngp*, *Ltf*, *Camp*, *Pglyrp1*, and *Ifitm3*) (37, 38), proinflammatory responses (*Pglyrp1*, *Wfdc21*, *Hmgb2*, and *S100a6*) (38, 39), cell migration, and reactive oxygen species production (*S100a6*, *Cybb*, *Cmss1*, and *Hmgb2*) (40, 41), were upregulated in *Sphk2^−/−^* neutrophils (**Figure 8B**). In contrast, genes involved in tissue repair, suppression of ROS (*Chil3*, *Mmp8*, *Malat*, *Trim30*, and *Trim12a*) (42–44), inhibition of neutrophil chemotaxis (*Arhgap15*) (45), inhibition of antigen presentation (*Stfa2* and *Stfa3*) (46), and anti-inflammatory response (*Chil3*, *Ifi208*, and *Trim30d*) (46, 47) were decreased in *Sphk2^−/−^* neutrophils (**Figure 8B**). These findings emphasize that the deletion of *Sphk2* alters the gene expression levels in bone marrow neutrophils, and hence neutrophil heterogeneity, upon Cl 13 infection.

To assess the distribution of the top DEGs in different neutrophil subsets, we classified neutrophils into P1–P4 subtypes based on their gene expression profiles, as described by Grieshaber-Bouyer et al. (48) (**Figure 8C**, **Supplementary Figure S12A**). These four subtypes represent highly proliferating P1 pre-neutrophils, immature P2–P3 neutrophils, and mature P4 neutrophils. Upon infection, mature neutrophils (P4) rapidly move into circulation; hence, their proportion decreases in the bone marrow. As shown in **Figure 8C**, a higher number of proliferating P1 neutrophils and fewer terminally differentiated mature P4 neutrophils were observed in *Sphk2^−/−^* mice (**Figure 8C**). Taken together, these results suggest elevated proliferation of neutrophil progenitors and increased egress of mature neutrophils into circulation, with eventual egress into peripheral organs. Next, we mapped the top DEGs for each subtype cluster to assess the distribution of the top DEGs within the subtypes (**Supplementary Figure S12B**). As shown in **Figure 8D**, genes involved in proinflammation were preferentially distributed in the P1–P2 subtypes, whereas anti-inflammatory and tissue repair-related genes were distributed in the P3–P4 subtypes, suggesting that phenotypic changes in neutrophils could occur as early as 3 dpi (**Supplementary Figure S12B**). Pathway analysis revealed changes in the neutrophil functional status of *Sphk2^−/−^* mice, including activation of the innate immune response to viral infection, interferon (IFN) signaling, oxidative damage response, decreased sphingolipid metabolism, TGF-β signaling, and apoptosis (**Figures 8E, F**). As the cellular response to the type I IFN pathway was noted to increase in SphK2-deficient neutrophils, gene signature profiling of the IFN pathway was further assessed (**Figure 8G**): 28 upregulated and 18 downregulated DEGs in the type I IFN system were identified in SphK2-deficient neutrophils. The majority (25 out of 28) of the upregulated genes were IFN-stimulated genes (ISGs). Genes encoding IFN signaling proteins, such as STAT2 and STAT1, were upregulated by SphK2 deficiency. In addition, three negative regulators of type I IFN production or signaling (*Oasl1*, *Usp18*, and *Smchd1*) were upregulated in SphK2-deficient neutrophils. Moreover, 10 IFN-related genes that can limit excessive inflammation, such as *Relb*, were downregulated in SphK2(-) neutrophils.. In summary, these results reinforce that SphK2-deficient neutrophils exhibit a proinflammatory phenotype during chronic LCMV infection and further support our *in vivo* experimental findings.

## Discussion

During chronic infection, suppressive neutrophils can develop and contribute to T cell dysfunction and viral persistence. Our study identified SphK2 as a host immune regulatory molecule crucial for neutrophil suppression during chronic LCMV infection. A key role of SphK2 in the regulation of neutrophil gene expression was uncovered, which supports the functional changes in neutrophils upon infection.

The depletion of Ly6G^+^ cells in WT mice during LCMV Cl 13 infection increased virus-reactive T cell immunity, contributing to a lower viral burden (**Figure 5**). These results indicate that Ly6G^+^ neutrophils acquire immunosuppressive functions upon infection, contributing to T cell dysfunction and LCMV persistence. Previous studies have depleted neutrophils using anti-Gr-1 antibody (RB6-8C5) during chronic LCMV Cl 13 infection (25). However, this antibody was reported to deplete other immune cells, including monocytes and eosinophils, in addition to neutrophils (49). Therefore, it was unclear whether neutrophils were indeed responsible for the regulation of T cell immunity caused by Gr1^+^ cell depletion in the previous study. As such, our study utilized anti-Ly6G antibody (1A8) treatment, which is known to specifically deplete neutrophils, to further demonstrate that neutrophils retain the immunosuppressive functions that impact T cell exhaustion and chronic LCMV infection. During acute infection, neutrophil numbers typically peak within the first 24 h and then gradually subside. However, LCMV Cl 13 spreads systemically and establishes viral persistence, accompanied by continuous neutrophil response.

SphK2 deficiency in mice did not affect the number of naïve T cells and neutrophils (**Supplementary Figure S13**). However, we observed an increase in virus-specific T cells (23) and neutrophils in multiple organs following LCMV infection. Following LCMV Cl 13-infection, SphK2-deficient neutrophils displayed a more pro-inflammatory phenotype, as evidenced by increased ROS production and decreased CD244 expression. The increased inflammatory neutrophilic response was shown to contribute to the viral immune pathogenesis of *Sphk2^−/−^* mice, as neutrophil depletion partially improved the viability of LCMV-infected *Sphk2^−/−^* mice. The reasons for the partial rescue of LCMV Cl 13 infected *Sphk2^−/−^* mice by neutrophil depletion are currently unknown and require further investigation. Neutrophils are a heterogeneous population, and not all of them are immune suppressors (50). It is possible that antibody-mediated depletion transiently removes all neutrophils, but some neutrophils may benefit virus clearance or tissue repair, thereby compromising the protective effects of depletion (51). Furthermore, immune cells other than neutrophils may also promote viral immune pathogenesis (23), and SphK2 may display immune regulatory functions in multiple immune cell types. However, although NK cells have been previously reported to contribute to T cell suppression during LCMV infection (8), NK cell number, frequency, and granzyme B^+^ NK cells did not change with SphK2 deficiency (**Supplementary Figure S14**), suggesting that NK cells are not important for the observed mortality of *Sphk2^−/−^* mice.

As both suppressive and immune-stimulatory neutrophils express many common phenotypic markers on their surfaces, it is difficult to differentiate them using flow cytometry. However, a few studies have used the SLAMF receptor CD244 to differentiate suppressive neutrophils from pro-inflammatory neutrophils (30, 31); targeted deletion of CD244 on myeloid cells improved CD8^+^ T cell effector functions (52, 53). We found a significantly reduced expression of CD244 in SphK2-deficient neutrophils during the early stage of infection (**Figure 3**, **Supplementary Figure S4**). Furthermore, the adoptive transfer experiment confirmed the neutrophil-intrinsic regulation of CD244 by SphK2 (**Figure 3F**). CD244, also known as 2B4 or SLAMF4, was also revealed to be a marker for T cell exhaustion, as its expression significantly increased in exhausted T cells during LCMV 13 infection (15). CD244 blockade along with anti-PDL-1 antibody treatment synergistically enhanced the antiviral response of CD8^+^ T cells upon LCMV Cl 13 infection, whereas CD244 blockade alone produced a moderate increase in CD8^+^ T cell functions (54). It is unclear whether the blockade of CD244 affects the function of suppressive neutrophils in previous studies. Blocking CD244 on pan-immune cells may not be a prudent approach because CD244 can act as either an inhibitory or stimulatory receptor depending on the cell type, receptor density, and availability of downstream intracellular signaling molecules (53). However, CD244 on neutrophils transmits inhibitory signals, suggesting that neutrophil-specific targeting of CD244 may be a better strategy.

Our scRNA-seq revealed differences in the neutrophil gene expression profile at the subpopulation level between WT and *Sphk2^−/−^* mice. Genes involved in opposing pathways (tissue repair vs. tissue damage) diverge in the early developmental stages. These subsets egress from the bone marrow at their current stage, after which they may develop into corresponding subtypes and perform their functions accordingly (55). *Chil3*, *Mmp8*, *Ifi208*, *Trim30d*, and *Trim12a*, which are involved in tissue remodeling and repair and are therapeutically targeted against inflammatory diseases in an experimental setting (56), were significantly increased in WT neutrophils (**Figure 8B**). Most importantly, *Ifi208*, *Trim30d*, and *Trim12a* were undetectable in *Sphk2^−/−^* neutrophils (**Figure 8D**, **Supplementary Figure S9B**). *Ifi208* (*Pydc3*) belongs to the interferon-inducible family p200 and inhibits inflammasome function (47). *Trim30* has been reported to inhibit cell proliferation in lymphoid cells (57), whereas in myeloid cells, it downregulates ROS production, NLRP3 inflammasome activation, and decreases the influx of neutrophils (58). *Trim12a* shares high sequence homology with *Trim30*; however, functional studies of this gene are very limited (59). Although a limited number of studies have demonstrated the immune regulatory functions of these genes, detailed studies on their regulation of neutrophil function are still needed. Furthermore, the possible importance of the type I IFN pathway in the stimulation of SphK2-deficient neutrophils and the resultant effects on immune regulation during infection remain to be investigated. Although the molecular mechanism by which SphK2 regulates neutrophils has not been reported, SphK2 has been shown to regulate gene expression in non-immune cells (21, 60). Therefore, SphK2 may directly control the expression of genes important for neutrophil suppression. Additionally, neutrophil populations generated during LCMV Cl 13 infection should be further characterized to define the function of SphK2 in neutrophil suppression and the consequent regulation of host immunity and virus clearance.

Identification of upstream or downstream targets of SphK2 in neutrophils may help us to regulate virus-specific T cell immunity more potently and consequent virus elimination. Thus, a better understanding of SphK2 regulation of neutrophil function will be helpful for designing advanced immune therapeutics against chronic infection as well as inflammatory conditions, which necessitates further studies on the role of SphK2 and its pathway during persistent viral infection associated with immune suppression or immunopathologic inflammation.

## Materials and methods

### Sex as a biological variable

We included both male and female C57BL/6 (WT) and C57BL/6 *Sphk2*^−/−^ mice in this study, and no differences between the sexes were observed; therefore, the results were pooled.

### Mice

All mice used in this study were male and female C57BL/6 (WT) (the Jackson Laboratory) and C57BL/6 *Sphk2*^−/−^ mice (provided by Richard Proia, NIH, Bethesda, Maryland, USA) aged between 6 and 10 weeks at the beginning of the study. Mice were either bred and maintained in a closed breeding facility according to institutional guidelines or purchased from the Jackson Laboratory. A maximum of five mice per cage were housed with *ad libitum* access to feed and water. At the end of the study, the mice were euthanized in a CO2 chamber according to the animal welfare law and institutional guidelines protocols approved by the Animal Care and Use Committee of the University of Missouri. In neutrophil depletion studies, animals that lost more than 25% of their initial body weight or were found to be moribund were euthanized in a small mouse cage (75 inch^2^ of floor space) with a 4 L/min flow rate of CO_2_. Animal studies were approved by the Animal Care and Use Committee of the University of Missouri-Columbia.

### Virus titration and infections

Lymphocytic choriomeningitis virus (LCMV) strains, LCMV Armstrong (Arm) and clone 13 (Cl 13), were propagated in BHK cells (61). LCMV titers were determined using plaque-forming unit (PFU) or focus-forming unit (FFU) assays on Vero E6 cells, as described elsewhere (23, 62). Mice were infected with 2 × 10^6^ FFU of LCMV Cl 13 via the intravenous (i.v.) route (63). For LCMV Arm experiments, mice were infected with 2 × 10^5^ FFU of LCMV Arm via the intraperitoneal (i.p.) route. Uninfected 6–8-week-old WT and *Sphk2^–/–^* mice were used as background controls in all *in vivo* experiments.

### Enrichment of bone marrow neutrophils

Hind legs were collected in ice-cold HBSS with 2% FCS and placed on ice for further analysis. All steps were performed aseptically in a laminar air chamber, and the samples were placed on ice. The samples were briefly dipped in 70% ethanol for 20 s–30 s. The femurs and tibias were separated, and the bone marrow was flushed with 1 ml of ice-cold HBSS + 2% FBS using a 26-gauage syringe. The debris were removed by passing the cell suspension through a 40 µm cell strainer. Cells were collected in a 50 ml falcon tube and centrifuged at 300×*g* at 4 °C for 5 min. RBCs were lysed by resuspending the pellet in 10 ml 0.2% NaCl for 20 s–25 s and neutralized by addition of equal volume (10 ml) 1.6% NaCl solution. Tubes were centrifuged as described above, and the pellet was resuspended in HBSS + 2% FBS buffer. To separate neutrophils from other mononuclear cells, 2 ml Histopaque 1119 (Sigma, St. Louis, MO) was taken in 15 ml falcon tube and on the top of this layer 3 ml Histopaque 1083 (Sigma, St. Louis, MO) was added slowly to the mixture. On top of this Histopaque gradient, 3 ml bone marrow cell suspension was gently overlaid and centrifuged at 872×*g* for 25 min at room temperature (RT). The upper mononuclear cell band was discarded, and the neutrophil-rich band present at the junction between Histopaque 1119 and 1083 was collected in 10 ml RPMI medium and washed twice, as mentioned above. The purity of the neutrophils was confirmed by neutrophil-specific cell surface marker staining using anti-mouse CD45, CD11b, and Ly6G antibodies by flow cytometry acquisition and analysis and used for downstream processing.

### Isolation of lymphocytes

Splenocyte suspensions were obtained by mashing the spleen through a 40 μm cell strainer. Cells were collected and pelleted by centrifugation at 300×*g* for 5 min at RT. RBCs were lysed using 1× RBC lysis buffer (Sigma), and cells were washed twice as mentioned above and finally resuspended in T cell medium (RPMI containing 10% FBS, 1% antibiotics, 1% non-essential amino acids, 1 mM HEPES, and 1 mM sodium pyruvate).

### Isolation of kidney cells

The kidney capsules were removed, chopped into small pieces, and transferred to a 5 ml serum-free RPMI medium containing 0.2 mg/ml collagenase D (Gibco) and 0.1 mg/ml DNase I (Sigma). The content was transferred to a gentle MACS dissociation C tube (Miltenyi Biotech) and homogenized according to the manufacturer’s protocol. The samples were incubated in a 37°C water bath for 30 min. The samples were again homogenized, passed through a 100 µm cell strainer, and centrifuged at 300×*g* for 5 min at RT. The pellet was resuspended in 1 ml RBC lysis buffer and incubated for 3 min at RT. RPMI medium was added to stop lysis, and the cells were centrifuged as described above. Cells were resuspended in HBSS containing 2% FBS, overlaid on 33% Percoll, and centrifuged at 300×*g* for 15 min at RT. The upper debris was removed, and the pellets were resuspended in RPMI medium and used for downstream processing.

### Neutrophil depletion

Neutrophils were depleted by administering 250 µg anti-Ly6G antibody (Clone 1A8, Leinco Technologies, St. Louis, MO) via the i.p. route. The control groups were treated similarly with the IgG isotype control antibody, IgG2a (Leinco Technologies, St. Louis, MO) on the indicated days, as described in the respective figure legends.

### Adoptive transfer of neutrophils

For neutrophil adoptive transfer experiments, WT and *Sphk2*^−/−^ mice were infected with LCMV Cl 13, and BMNs were isolated at 3 dpi, as described above. The purity (80%–90%) of neutrophils was assessed by flow cytometry prior to the adoptive transfer of 2 × 10^6^ neutrophils into WT mice via i.v. injection on the indicated days.

### Adoptive transfer of CFSE-stained neutrophils

BMNs were isolated from uninfected WT and Sphk*2*^−/−^ mice and incubated with 5 µg/ml CFSE (Thermo Fisher) at 37°C in the dark for 15 min. The cells were then washed twice with RPMI medium and resuspended in 1× DPBS. Subsequently, these CFSE-stained neutrophils (2 × 10^6^) were adoptively transferred to LCMV Cl 13-infected wild-type mice at 3 dpi. After two days of transfer, spleen and kidney samples were collected to assess the expression of CD244 on the adoptively transferred donor neutrophils in the recipient mice.

### Reactive oxygen species quantification

BMNs were isolated from LCMV Cl 13-infected WT and *Sphk2*^−/−^ mice at 3 dpi. Cells (1 × 10^5^ cells/well) were seeded in a 96-well plate and cultured in a CO_2_ incubator for 30 min at 37°C, in the presence or absence of 50 nM PMA. The cells were washed and resuspended in 100 µL of FACS staining buffer containing 5 µM H2DCFDA. The plate was incubated at 4°C for 30 min in the dark. Cells were washed twice with FACS staining buffer, and samples were acquired using an LSR Fortessa X420 flow cytometer. Mean fluorescence intensity (MFI) was analyzed using FlowJo software. ROS production was measured in a time-dependent manner using luminol-enhanced chemiluminescence assay. Briefly, 1 × 10^5^ cells/well were seeded in 96 well plate and cultured in a CO_2_ incubator for 15 min at 37°C in the presence of 5 µM luminol. After incubation, 50 nM PMA was added to the designated wells, and intracellular chemiluminescence was read from the top of each well at 2 min intervals for 40 min using a microplate reader (CLARIOstar^®^ Plus, BMG Labtech).

### Flow cytometric analysis

Surface antibody staining was performed on a 96-well V-bottomed plate. Cells (1 × 10^6^ cells/well) were resuspended in 50 µL of FACS staining buffer containing the appropriate antibody. For flow cytometry, singlet cells were selected and CD45 positive leukocytes were used for further analysis. LCMV GP_33–44_-specific CD8^+^ T cells were identified using fluorochrome-linked GP_33–41_ tetramers, and LCMV GP_66–77_-specific CD4^+^ T cells were identified using fluorochrome-linked GP66 tetramers provided by the NIH Tetramer Core Facility (Emory University, Atlanta, Georgia, USA). For intracellular cytokine staining, lymphocytes were cultured in the presence of 10 μg/mL of brefeldin A (Sigma, St. Louis, MO, USA) and 2 μg/mL GP33 (KAVYNFATC) and 5 μg/mL GP_61–80_ (GP61, GLNGPDIYKGVYQFKSVEFD) peptides for 6 h, followed by fixation, permeabilization, and staining with the indicated antibodies. Samples were run on LSR Fortessa X-20 (BD Biosciences) or Cytek Aurora spectral analyzers (Cytek Biosciences) and analyzed using FlowJo software. The antibodies used in this study are listed in the **Supplementary Data Sheet 2.**.

### Determination of virus titers

Tissues from the liver, kidney, and spleen, along with serum, were harvested from infected mice at the indicated time. Tissues were homogenized using a Bead Beater with 1.0 mm diameter zirconia/silica beads (BioSpec Products). LCMV titers were determined using the FFU or PFU assay on Vero cells, as previously described (23).

### Single-cell RNA-sequencing and bioinformatic analysis

Bone marrow was collected from uninfected WT and LCMV Cl 13-infected WT and *Sphk2*^−/−^ mice at 3 dpi. Bone marrow cells were isolated as mentioned above, with minor modifications in the isolation and suspension buffers. Cells were passed through a 40 µm cell strainer twice to remove debris and aggregates. Total bone marrow cell suspensions were resuspended in PBS with 0.04% BSA. Finally, 1 × 10^6^ cells/ml with >85% viable bone marrow cell suspensions were prepared and immediately handed over to the university DNA core facility for scRNA-seq.

### 10× Genomics single cell 3’ RNA-seq library preparation method

Libraries were constructed following the manufacturer’s protocol with reagents supplied in the 10× Genomics Chromium Next GEM Single Cell 3′ Kit v3.1. Briefly, cell suspension concentration and viability were measured using a Cellometer K2 (Revvity) stained with an acridine orange/propidium iodine dye mix (Invitrogen). Subsequently, the cell suspension (targeting 10,000 cells) combined with reverse transcription master mix was loaded onto a Chromium Next GEM chip G along with gel beads and partitioning oil to generate gel emulsions (GEMs). GEMs were then transferred to a PCR strip tube, followed by reverse transcription, which was performed on a Veriti thermal cycler (Applied Biosystems) at 53 °C for 45 min. cDNA was amplified for 11 cycles and purified using AxyPrep MagPCR Clean-up beads (Axygen). cDNA fragmentation, end-repair, A-tailing, and ligation of sequencing adaptors were performed according to the manufacturer’s specifications. Library concentration was measured using a Qubit HS DNA kit (Invitrogen), and the fragment size was measured using a 5200 Fragment Analyzer (Agilent). Libraries were pooled and sequenced on a NovaSeq X (Illumina) to generate 50,000 reads per cell with a sequencing configuration of 28 base pairs (bp) on read 1 and 100 bp on read 2.

### scRNA-seq data processing

Single-cell RNA sequencing (scRNA-seq) data from mouse bone marrow were processed using the CellRanger toolkit (v8.0.1) with alignment to the GRCm39 mouse reference genome. Only reads that were uniquely aligned, non-PCR duplicates, and associated with valid cell barcodes and unique molecular identifiers (UMIs) were used to construct the initial 3′ gene-by-cell matrix, which comprised 30,500 cells.

To correct for ambient RNA contamination and barcode swapping, we applied CellBender (v0.3.2) (64). Cells were retained if they passed a false discovery rate (FDR) threshold of <0.01, adjusted using the Benjamini–Hochberg method. Additional quality filters were imposed to exclude low-quality cells: (1) total UMI count >1,000, (2) number of detected genes >500, and (3) proportion of mitochondrial transcripts <10%.

Potential doublets were filtered using a cluster-level approach. DoubletDetection (v4.3) was used to compute the doublet score for each cell (65). Cells were grouped into clusters per sample by computing the top 50 principal components from the 3,000 most variable genes, followed by the construction of a k-nearest neighbor graph via the pp.neighbors function and clustering using the Louvain algorithm implemented in Scanpy (v1.11.0) (66, 67). Median-centered, MAD-scaled doublet scores were calculated for each cluster to identify and remove the likely doublets.

After all quality control procedures, 19,004 high-confidence cells from three biological samples (nine mice) were retained for downstream analysis. For each retained cell, both raw UMI counts and log_2_-transformed normalized expression values were calculated.

### Integrated analysis of single-cell datasets

To account for inter-sample variability and batch-associated confounders, we utilized the scVI-tools framework to perform an integrated analysis of single-cell transcriptomes (68). This approach applies single-cell variational inference (scVI), a deep generative modeling technique built upon variational autoencoders (VAEs), to learn a unified latent representation of gene expression profiles, while controlling batch effects.

In the scVI model, each cell is encoded into a low-dimensional latent variable conditioned on known batch annotations. The generative decoder then reconstructs the observed expression levels using this latent embedding combined with the batch covariate. By explicitly incorporating the batch as a covariate during training, scVI disentangles biological variation from technical artifacts and captures the intrinsic structure of the data across samples.

During model inference, batch labels can be marginalized to yield batch-harmonized latent embeddings, which support integrated downstream analyses, such as dimensionality reduction, clustering, and trajectory inference. These corrected representations offer a probabilistically grounded and scalable solution for batch correction and are particularly well-suited for large and heterogeneous single-cell datasets.

### Visualization and signature-based cell classification

Major immune cell types were identified using the Scanpy Python package. We first selected 3,000 highly variable genes (HVGs), followed by principal component analysis (PCA) to compute the top 50 components for reducing dimensionality. Prior to downstream analysis, the percentage of mitochondrial transcripts was regressed out, and all gene expression values were scaled to unit variance. A k-nearest neighbor graph was constructed using the pp.neighbors function, and clustering was performed using the Leiden algorithm at a resolution of 1, resulting in 26 transcriptionally distinct clusters. The structure of the cellular landscape was visualized using UMAP for a two-dimensional embedding.

Initial annotation of major immune cell types was performed using SingleR (v2.10.0) (69), referencing the MouseRNAseqData database from Celldex (v1.18.0). To further refine these annotations, we computed gene signature scores using the tl.score_genes function in Scanpy, based on canonical marker genes. Neutrophils were characterized by high expression of *Ly6g*, *Itgam*, *Cxcr2*, *Csf3r*, *S100a8*, *Il1r2*, *Trem1*, *Ceacam1*, *Hp*, and *Hdc*. Neutrophils were defined as cells exhibiting *Ly6g* expression greater than zero and a positive neutrophil gene module score. Macrophages expressed *Mrc1*, *Cd68*, and *Adgre1*, whereas monocytes were marked by *Plac8*, *Psap*, and *Ccr2*. Dendritic cells were identified by the expression of *Km0* and *Flt3*. T cells were annotated based on *Cd3d*, *Cd3e*, and *Trbc2*, whereas B cells were annotated based on *Cd79a*, *Cd79b*, and *Pxk*. Natural killer cells showed high levels of *Klrd1*, *Klrk1*, and *Nkg7*, and granulocytes were distinguished by *Csf3r*, *Clec4d*, and *Cxcr2* expression.

To investigate transcriptional heterogeneity within the neutrophil population, we conducted a second round of clustering exclusively on neutrophil-annotated cells. Gene module scores were calculated for subpopulation classification based on curated markers as described by Grieshaber-Bouyer et al. (48). One subpopulation, designated as p1, was defined by the elevated expression of *Cebpe*, *Hmgb2*, *Chil3*, *Ngp*, *Arhgdib*, *Calm2*, *mt-Co1*, *Lcn2*, *Lyz2*, *Wfdc21*, *Cybb*, *Ly6c2*, *Ly6g*, *Cd177*, *Serpinb1a*, *Pglyrp1*, and *Prdx5*. A second group, p2, was enriched for *Ltf*, *Lcn2*, *Lyz2*, *Wfdc21*, *Ifitm6*, *Anxa1*, *Mmp8*, *Cybb*, *Dstn*, *Ly6c2*, *Ly6g*, *Cd177*, *Prdx5*, *Lgals3*, *Mmp9*, *Pglyrp1*, and *Mcemp1*. The third subpopulation, p3, showed elevated levels of *Mmp8*, *Lgals3*, *Mcemp1*, *Retnlg*, *S100a6*, *Prr13*, *Fth1*, *Ccl6*, *Msrb1*, and *H2-D1*. Finally, the p4 subset was characterized by the expression of *Wfdc17*, *Ifitm1*, *Ifitm2*, *Btg1*, *Fxyd5*, *Srgn*, *Malat1*, *Dusp1*, *Rps27*, *Jund*, *Fth1*, *Msrb1*, *Csf3r*, *Junb*, and *H2-D1*.

We identified eight major immune cell types and four transcriptionally distinct neutrophil subpopulations. Differentially expressed genes across clusters and neutrophil subsets were identified using the tl.rank_genes_groups function in Scanpy, applying the Wilcoxon rank-sum test, and controlling the false discovery rate using the Benjamini–Hochberg procedure. Genes with an adjusted P-value <0.05 were considered statistically significant. The complete and detailed scRNA-seq data can be found in the GEO database (GSE303763) GEO Accession viewer.

### Pathway enrichment analysis

To investigate the functional relevance of genes identified as cluster-specific markers, we performed gene set enrichment analysis using GSEApy (v1.1.8) (70). Differential expression results from the *Sphk2(+)* neutrophil population under WT treatment were used to define the gene sets of significantly upregulated and downregulated transcripts. These gene sets were queried against multiple curated databases, including Enrichr, the Gene Ontology (GO) Biological Process, and WikiPathways signaling collections (71). This analysis enabled the identification of biological processes and signaling pathways significantly associated with the transcriptional response of neutrophils to treatment, providing insights into the molecular programs regulated by Sphk2 activity.

### Statistical analysis

All error bars represent the mean ± SEM, and averages were compared using a bidirectional (2-tailed), unpaired Student’s t-test unless otherwise indicated. In the case of different sample sizes, an unequal variance t-test was employed. For viral titers, an unpaired two-tailed t-test was used to account for nonparametric viral clearance. Graphs were constructed and statistical analyses were performed using Prism 10 (GraphPad). For time-dependent ROS production between wild-type and *Sphk2^−/−^* mice, a 2-way ANNOVA test was performed. For the mouse survival study, the Kaplan–Meier test was used to determine the statistical significance between different treatments. A p-value less than or equal to 0.05 was considered statistically significant for this analysis. For scRNA-seq data, a q-value (FDR) of less than 0.05 was used, whereas for GSEA pathway analyses, a q-value (FDR) of less than 0.1 was considered significant.

## Data Availability

The datasets presented in this study can be found in online repositories. The names of the repository/repositories and accession number(s) can be found in the article/**Supplementary Material**.

## Supplementary material

Supplementary Figure 1 Monocyte and macrophage populations do not change in blood of *Sphk2^-/-^* mice upon LCMV infection. WT and *Sphk2^-/-^* mice (n = 3-5/group) were uninfected or infected with LCMV Cl 13. Mice were euthanized at 3 dpi or 8 dpi, and blood was collected. The proportions of monocytes (CD11b^+^Ly6C^+^) **(A)** and macrophages (CD11b^+^F4/80^+^) **(B)** out of CD45^+^ cells were assessed by flow cytometry. ns. not significant, bidirectional, unpaired Student’s *t*-test.

Supplementary Figure 2 LCMV Cl 13 infection induces enhanced and sustained neutrophils in *Sphk2^-/-^* mice. WT and *Sphk2^-/-^* mice (n = 5/group) were infected with LCMV Cl 13. Mice were euthanized at 14 dpi, and organs were collected. The percentage of CD11b^+^Ly6G^+^ neutrophils in bone marrow (BM), spleen, and blood of LCMV Cl 13-infected WT and *Sphk2^-/-^* mice was quantified using flow cytometry. **p ≤ 0.01, ns, not significant, bidirectional, unpaired Student’s *t*-test.

Supplementary Figure 3 LCMV Cl 13 induces higher neutrophils compared to LCMV Arm. WT (n = 5/group) were infected with LCMV Arm or Cl 13. At 10 dpi, mice were euthanized and splenocytes were isolated. The percentage of CD11b^+^Ly6G^+^ neutrophils from uninfected control, LCMV Arm and Cl 13 infected group was quantified by flow cytometry **(A)**. The representative flow cytometric dot plot of one sample from each group is depicted **(B)**. ****p ≤ 0.0001, ns, not significant. 1-way ANOVA was performed to find the significant difference between the groups.

Supplementary Figure 4 Neutrophil depletion in *Sphk2*^-/-^ mice by the treatment with anti-Ly6G antibody. Depletion of neutrophils was performed by injecting *Sphk2^-/-^* mice with 250 µg anti-Ly6G antibody (αLy6G) or control-antibody (αIgG) via the intraperitoneal (i.p.) route one day before and one day after LCMV Cl 13 infection. At 3 dpi and 7 dpi, 3 mice per group were analyzed to evaluate neutrophil depletion efficiency by quantifying the frequency of neutrophils in peripheral blood. Representative flow cytometric dot plot of one sample from each group is shown.

Supplementary Figure 5 LCMV Cl 13 infection induces significantly increased CD244^+^ neutrophils in different organs. WT mice (n = 5-6) were uninfected (control) or infected with LCMV Cl 13, and at 3 dpi mice were euthanized and organs were collected. The percentage of CD244^+^ neutrophils in different organs was quantified by flow cytometry, and the percentage levels were compared with those of uninfected C57BL/6 WT mice. ****p ≤ 0.0001, ***p ≤ 0.001, **p ≤ 0.01, bidirectional, unpaired Student’s *t*-test.

Supplementary Figure 6 SphK2 deficiency reduces the expression levels of CD244 on neutrophils during LCMV Cl 13 infection. WT and *Sphk2*^-/-^ mice (n = 5/group) were infected with LCMV Cl 13, and at 3 and 8 dpi, mice were euthanized, and organs were collected. The geometric mean fluorescence intensity (MFI) of CD244 on neutrophils in multiple organs at 3 dpi **(A)** and 8 dpi **(B)** were measured by flow cytometry, and the expression level of CD244 were compared between LCMV Cl 13 infected WT and *Sphk2*^-/-^ neutrophils. ***p ≤ 0.001, **p ≤ 0.01, *p ≤ 0.05, bidirectional, unpaired Student’s *t*-test.

Supplementary Figure 7 Production of ROS is not different between uninfected WT and *Sphk2^-/-^* neutrophil. Bone marrow neutrophils were isolated from uninfected C57BL/6 and *Sphk2^-/-^* mice (n = 4). BMNs were cultured in the absence **(A)** or presence of 50 nM PMA for 30 min at 37^°^C in a CO2 incubator **(B)**, and production of intracellular ROS was detected by incubating the cells with H2DCFDA for 30 min at 4^°^C in the dark. The mean fluorescence intensities (MFIs) of H2DCFDA were detected by flow cytometry. ns, not significant, bidirectional, unpaired Student’s *t*-test.

Supplementary Figure 8 Anti-Ly6G antibody treatment depletes the neutrophils. WT mice (n = 6/group) were infected with LCMV Cl 13 and were treated with i.p. injection of mice with 250 µg IgG isotype control or anti-Ly6G antibody (clone 1A8). 3 days post antibody treatment, 50 µl venous blood was collected from both groups and the percentage of neutrophils (CD11b^+^Ly6G^+^ cells) was quantified by flow cytometry.

Supplementary Figure 9 Quantifying the purity of enriched bone marrow neutrophils (BMNs). Bone marrow neutrophils were isolated from WT and *Sphk2^-/-^* mice as described in the method section. The purities of enriched BMNs (CD11b^+^Ly6G^+^) were quantified by flow cytometry.

Supplementary Figure 10 Adoptively transferred *Sphk2^-/-^* neutrophils reduce virus burden. BMNs from LCMV Cl 13 infected WT or *Sphk2^-/-^* mice (n = 6/group) were adoptively transferred to LCMV Cl 13 infected recipient mice (n=6/group) at 3 dpi, 5 dpi, 12 dpi, and 19 dpi. The virus titers in the serum at 25 dpi **(A)** and 32 dpi **(B)**, liver at 32 dpi **(C)**, and kidney at 32dpi **(D)** were measured by FFU assay. **p ≤ 0.01, *p ≤ 0.05, ns, not significant, bidirectional, unpaired Student’s *t*-test.

Supplementary Figure 11 Annotation of bone marrow cell types. Bone marrow cell types were annotated based on the expression of cell-specific marker gene or combination of gene expression profiles of specific cell subtypes.

Supplementary Figure 12 Neutrophil subtyping and distribution of top 30 DEGs on neutrophil subtypes. Bone marrow neutrophils were classified into P1-P4 subtypes based on their gene expression profile **(A)**. Comparison top 30 DEGs distribution on different subtypes of neutrophils between WT and *Sphk2^-/-^***(B)**.

Supplementary Figure 13 Naïve T cells and neutrophils are not affected by the deletion of SphK2. Spleenocytes from uninfected WT or *Sphk2^-/-^* mice (n = 4-5/group) were collected. Frequency and absolute number of naïve CD4^+^ T cells **(A)** and CD8^+^ T cells **(B)** were assessed by flow cytometry. The frequency and number of neutrophils is shown **(C)**. ns, not significant, bidirectional, unpaired Student’s *t*-test.

Supplementary Figure 14 NK cells are not affected by the deficiency of SphK2 during LCMV infection. WT and *Sphk2*^-/-^ mice (n = 4-5/group) were uninfected or infected with LCMV Cl 13, and spleens were collected at 3 dpi. Frequency and absolute number of NK (NK1.1^+^) cells **(A, B)** and GZMB^+^ NK cells **(C, D)** were assessed by flow cytometric analysis. ns, not significant, 1-way ANOVA analysis was performed to evaluate significant differences between the groups.
