# Supplementary material for: Sphingosine kinase 2 suppresses neutrophil responses to promote viral persistence while attenuating immune pathology
Source: Front Immunol. 2026 Jan 22;16:1706967. doi: 10.3389/fimmu.2025.1706967 (PMC12872563; doi:10.3389/fimmu.2025.1706967)

Supplementary Figures

Fig. S1. Monocyte and macrophage populations do not change in blood of *Sphk2$^{-/-}$* mice upon LCMV infection.

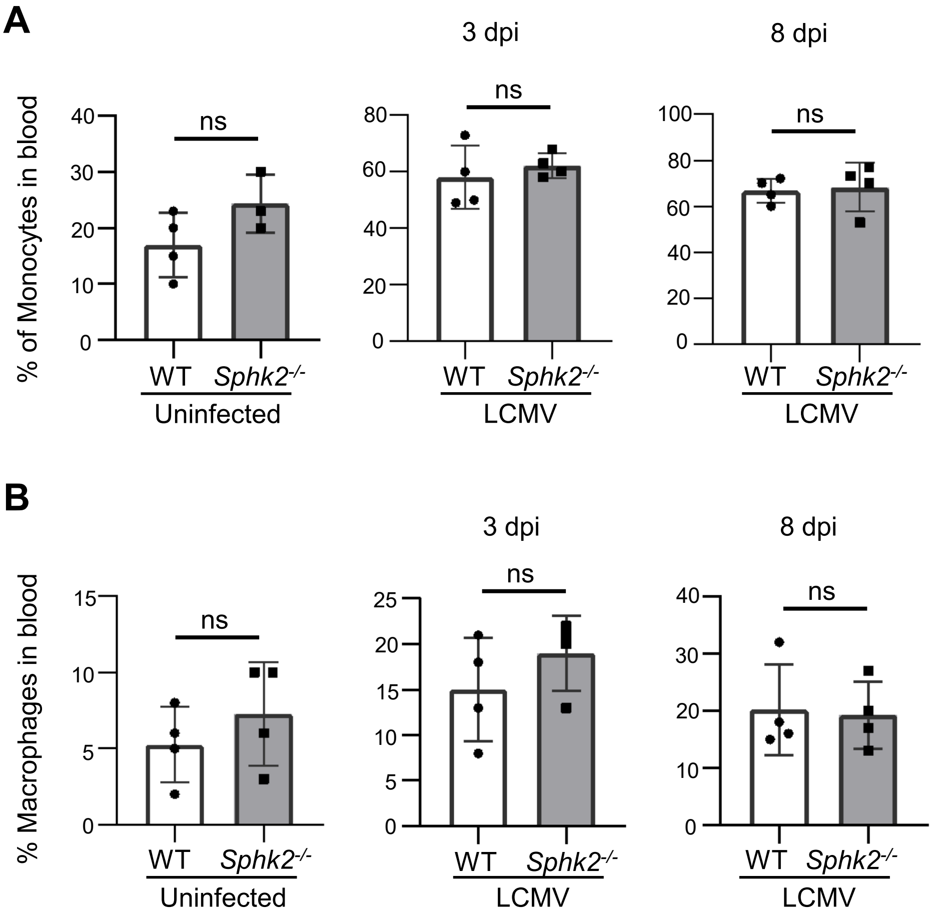

Fig. S2. LCMV Cl 13 infection induces enhanced and sustained neutrophils in *Sphk2*$^{-/-}$ mice.

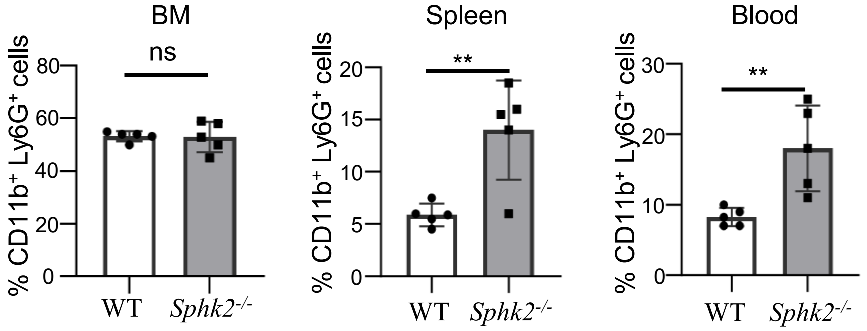

Fig. S3. LCMV Cl 13 induces higher neutrophils compared to LCMV Arm.

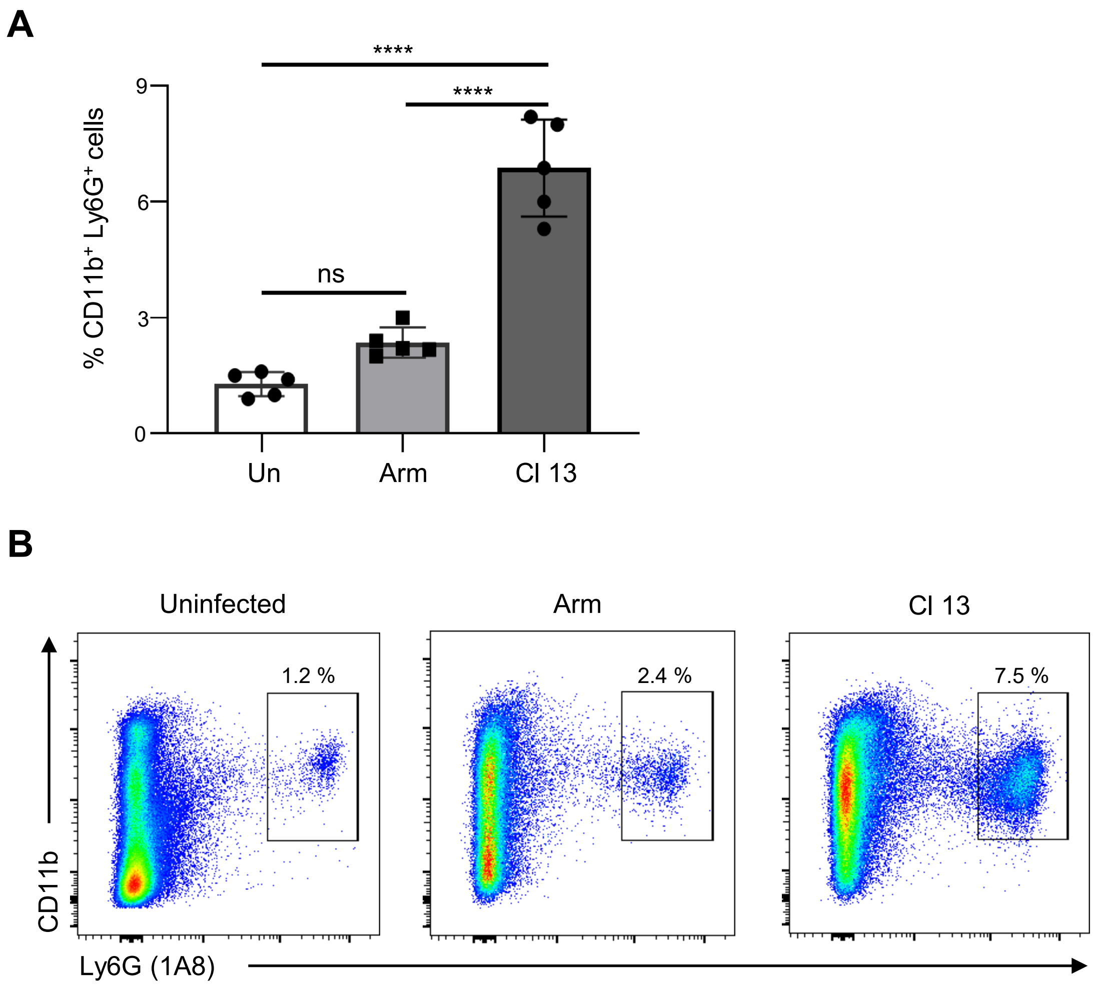

Fig. S4. Neutrophil depletion in *Sphk2*$^{-/-}$ mice by the treatment with anti-Ly6G antibody.

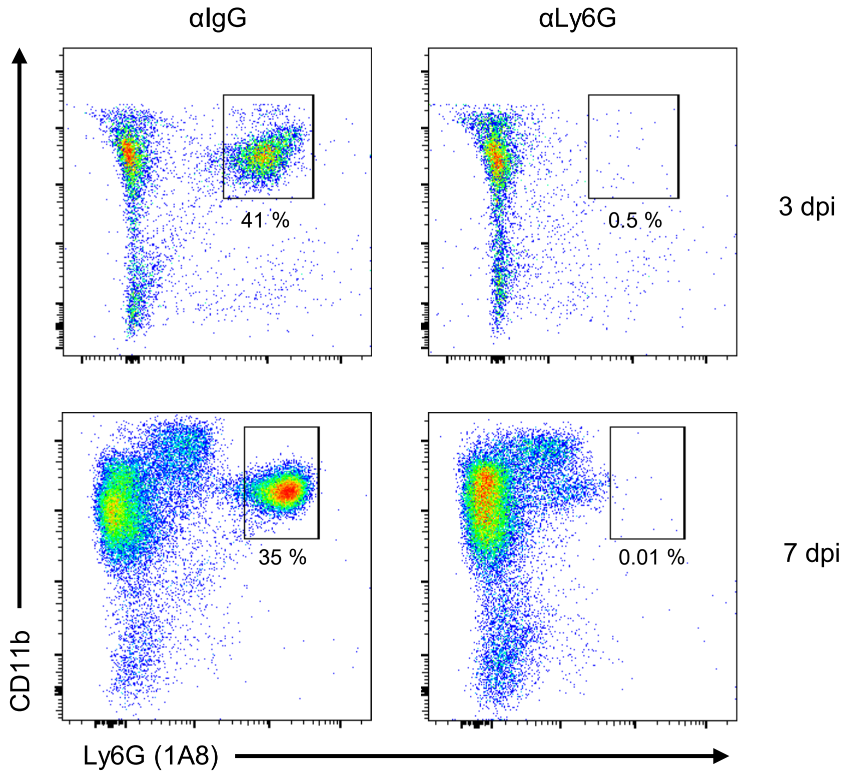

Fig. S5. LCMV Cl 13 infection induces significantly increased CD244$^+$ neutrophils in different organs.

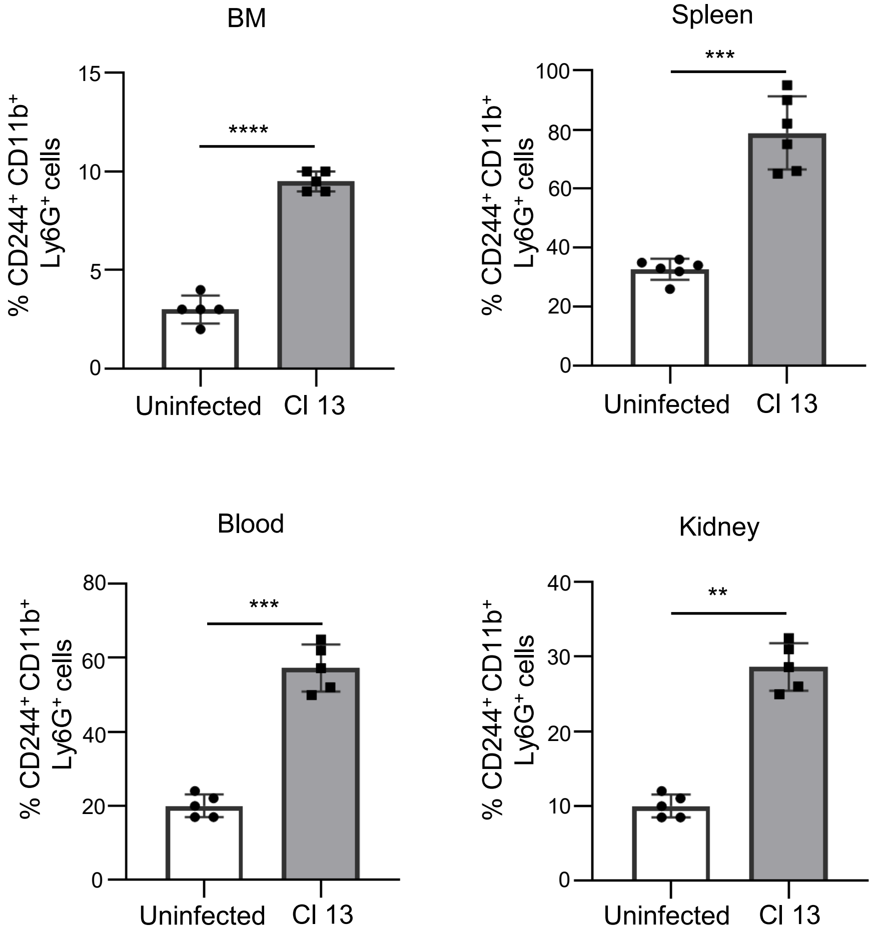

Fig. S6. SphK2 deficiency reduces the expression levels of CD244 on neutrophils during LCMV Cl 13 infection.

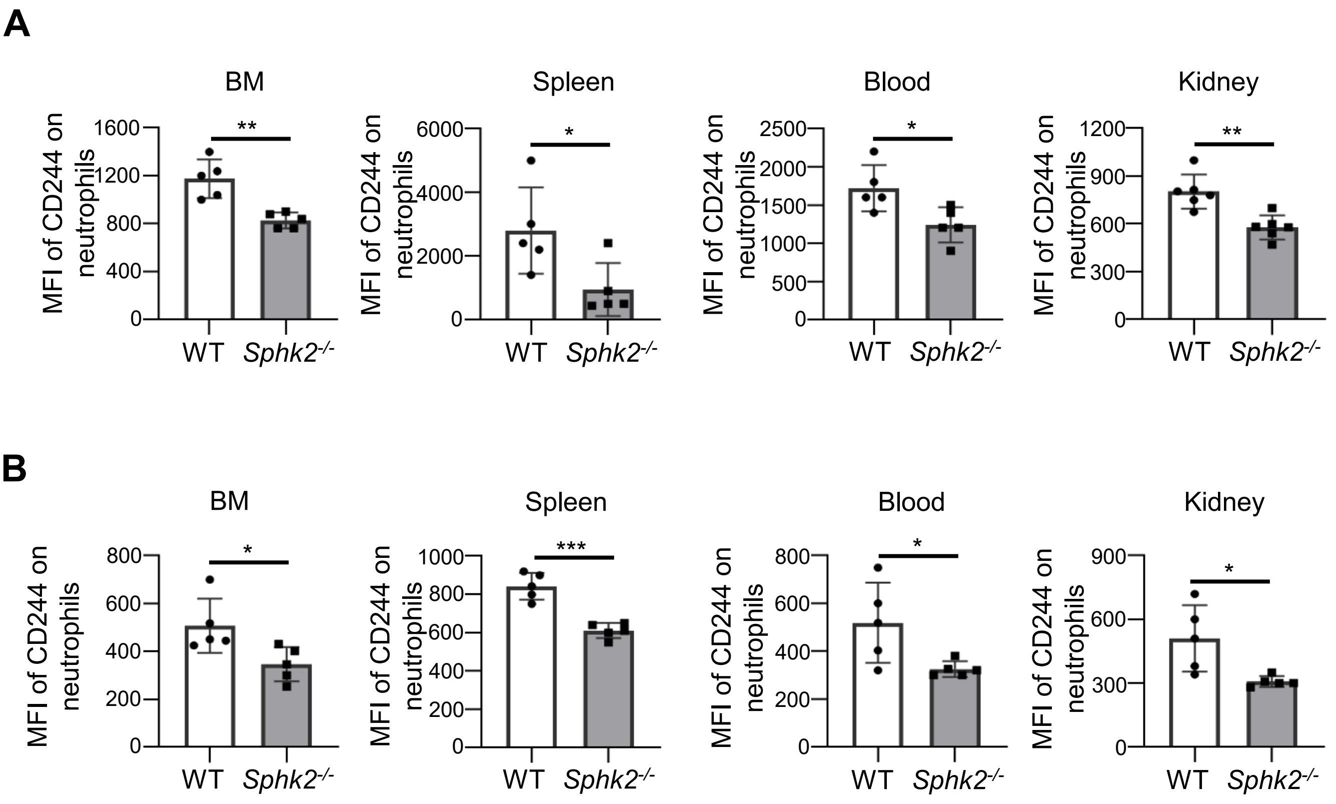

Fig. S7. Production of ROS is not different between uninfected WT and *Sphk2*$^{-/-}$ neutrophil.

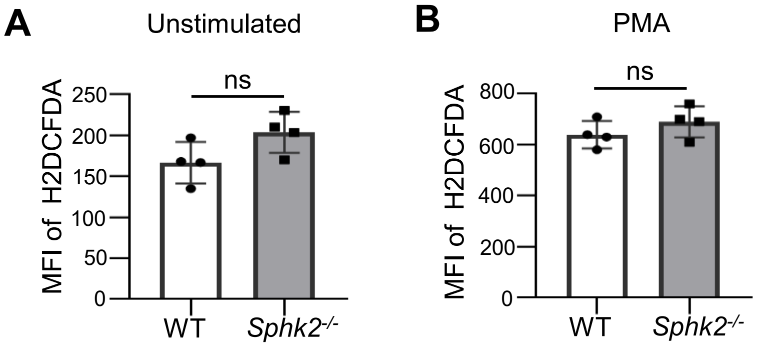

Fig. S8. Anti-Ly6G antibody treatment depletes the neutrophils.

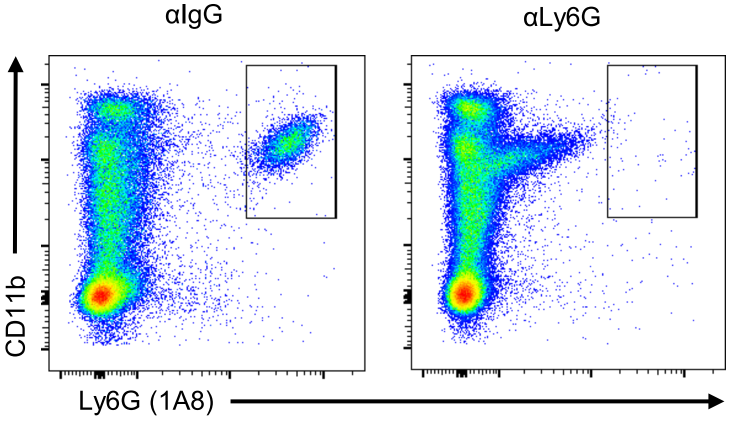

Fig. S9. Quantifying the purity of enriched bone marrow neutrophils (BMNs).

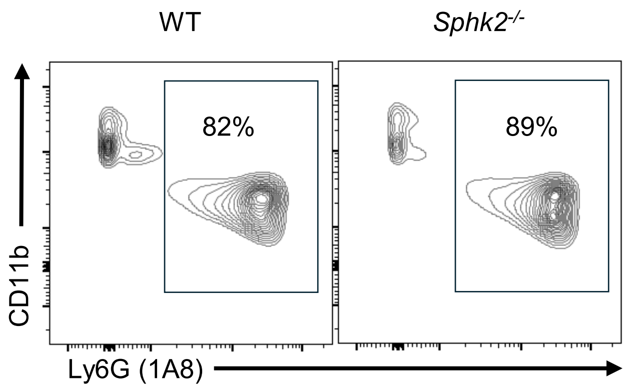

Fig. S10. Adoptively transferred *Sphk2*$^{-/-}$ neutrophils reduce virus burden.

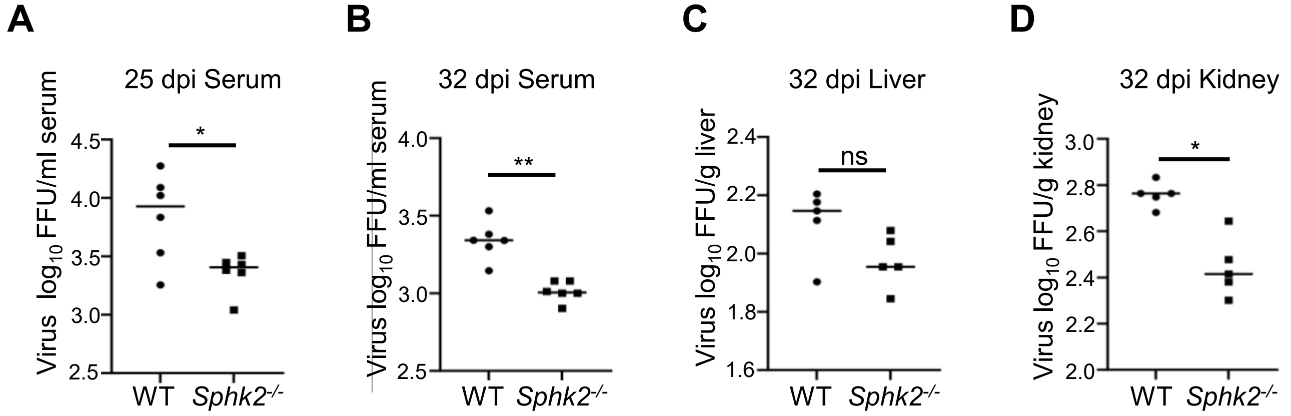

Fig. S11. Annotation of bone marrow cell types.

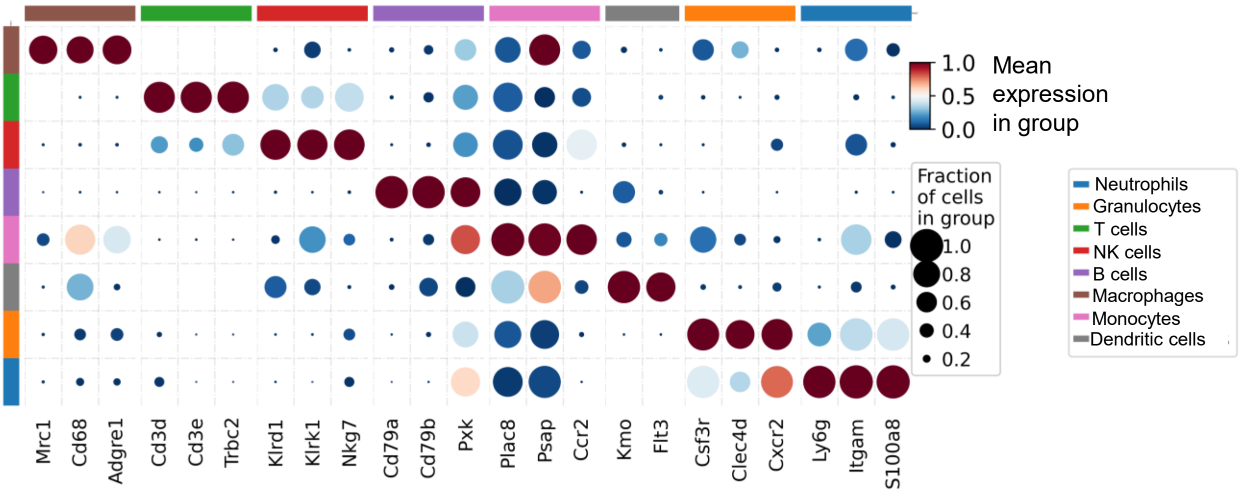

Fig. S12. Neutrophil subtyping and distribution of top 30 DEGs on neutrophil subtypes.

A

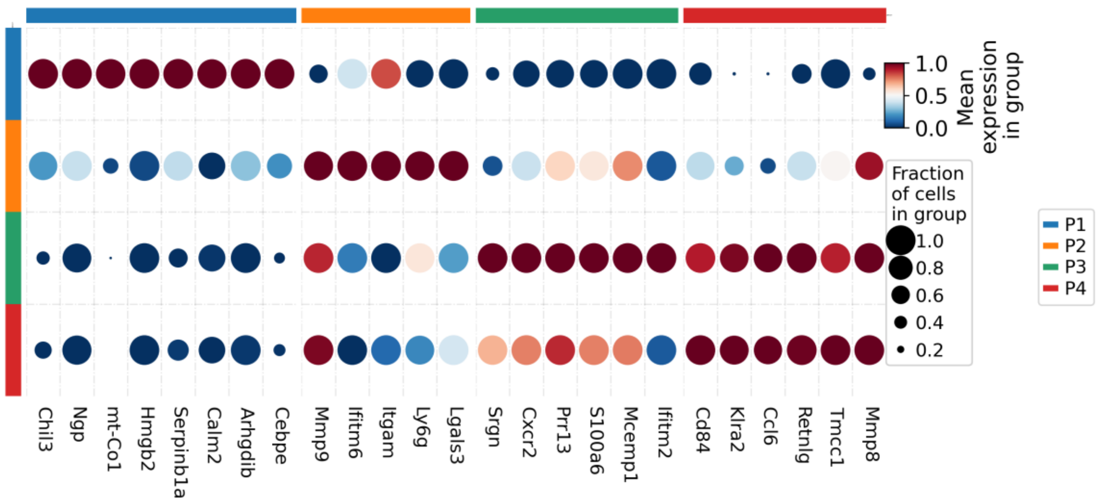

B

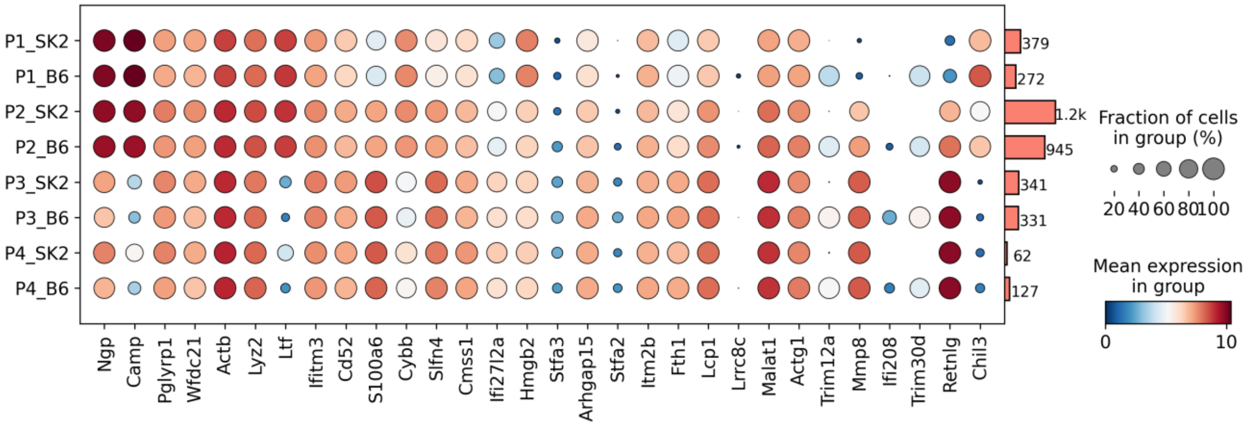

Fig. S13. Naïve T cells and neutrophils are not affected by the deletion of SphK2.

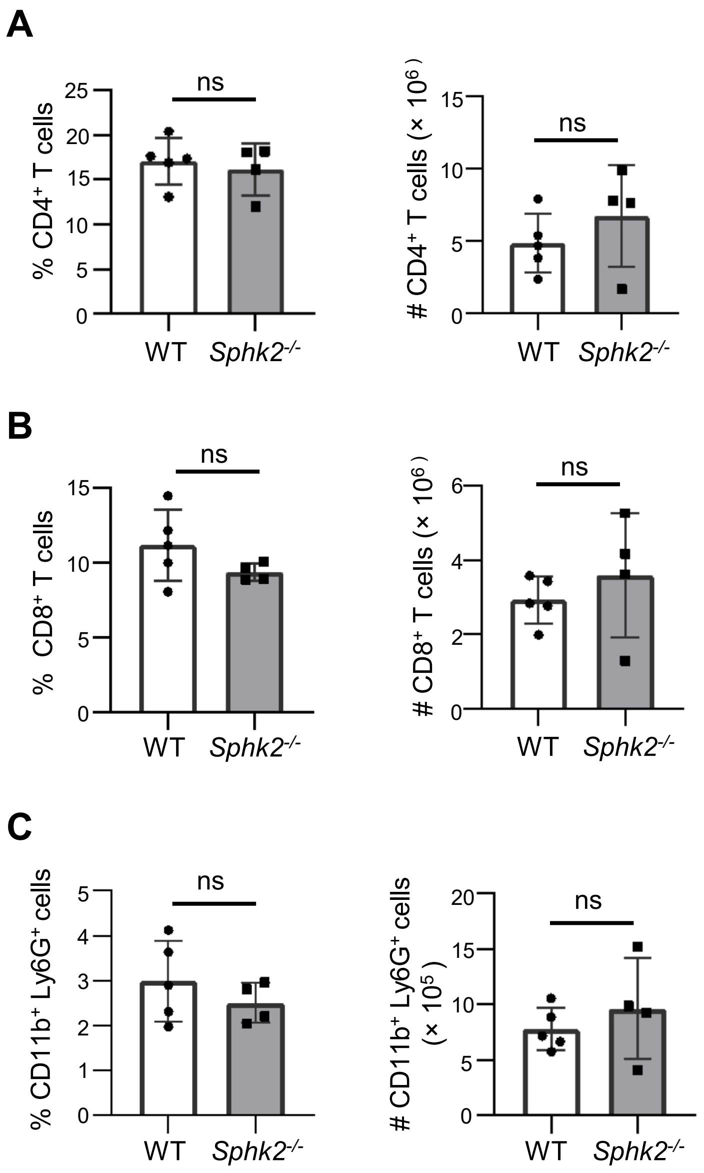

Fig. S14. NK cells are not affected by the deficiency of SphK2 during LCMV infection.

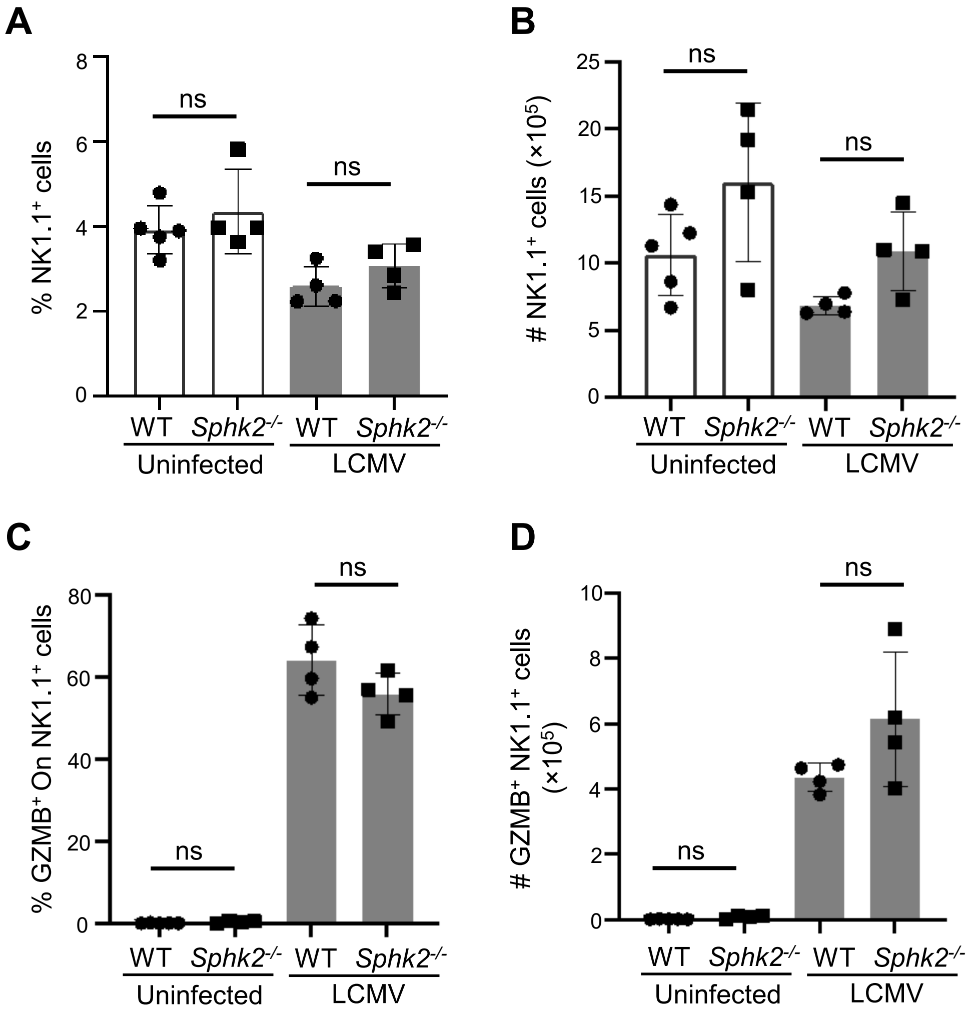

Supplement: Supplementary Figure 1 — Monocyte and macrophage populations do not change in blood of Sphk2-/- mice upon LCMV infection. WT and Sphk2-/- mice (n = 3-5/group) were uninfected or infected with LCMV Cl 13. Mice were euthanized at 3 dpi or 8 dpi, and blood was collected. The proportions of monocytes (CD11b+Ly6C+) (A) and macrophages (CD11b+F4/80+) (B) out of CD45+ cells were assessed by flow cytometry. ns. not significant, bidirectional, unpaired Student’s t-test. [file DataSheet1.pdf]
