# Supplementary material for: Sphingosine kinase 2 suppresses neutrophil responses to promote viral persistence while attenuating immune pathology
Source: Front Immunol. 2026 Jan 22;16:1706967. doi: 10.3389/fimmu.2025.1706967 (PMC12872563; doi:10.3389/fimmu.2025.1706967)
Supplement: Supplementary file 2 [file DataSheet2.pdf]

Supplementary table S1: Antibodies used in this study.

Antibody	Clone	Maker	Catalog
CD45-BV785	30-F11	Biolgened	103147
CD11b-PerCPCy5.5	M1/70	Biolegend	101230
Ly6G-APC	1A8	Biolegend	127614
Ly6C-PE	HK1.4	Biolegend	128008
Ly6C-BV605	HK1.4	Biolegend	128036
CD244-PECy7	m2B4	Biolegend	133511
CD244-FITC	m2B4	Biolegend	133503
Ly6C-FITC	HK1.4	Biolegend	128005
CD3-PerCPCy5.5	17A2	Biolegend	100218
CD8-FITC	5H10-1	Biolegend	100804
CD4-FITC	RM4-5	Biolegend	100510
CD45-FITC	S18009F	Biolegend	157214
Ly6G-BV421	1A8	Biolegend	127627
CD19-APC	6D5	Biolegend	115511
IFN-γ-PE	XMG1.2	BD Bioscience	554412
CD3-PE	KT3.1.1	Biolegend	155607
MHC-II-PE	AF6-120.1	BD Bioscience	553552
Tim-3	B8.2C12	Biolegend	134010
PD-1-BV785	29F.1A12	Biolegend	135225
CD8-BV605	53-6.7	Biolegend	100744
TNF-α-APC	MP6-XT22	Biolegend	506307
Granzyme B-PE	QA18A28	Biolegend	396405
Granzyme B-APC	QA18A28	Biolegend	396408
Ki67-FITC	16A8	Biolegend	652410
CD4-BV605	RM4-5	Biolegend	100548
TNF-α-BV605	MP6-XT22	Biolegend	506329
NK1.1-PECy5	S17016D	Biolegend	156523
F4/80-PE	W20065B	Biolegend	111603
InVivoMAb anti-LCMV nucleoprotein	VL-4	BioXcell	BE0106
Goat anti-Rat IgG (H+L) Secondary Antibody		Invitrogen	31470
